# Exosome-Based Smart Drug Delivery Tool for Cancer Theranostics

**DOI:** 10.1021/acsbiomaterials.2c01329

**Published:** 2023-01-09

**Authors:** Rishav Kar, Rajib Dhar, Sayantanee Mukherjee, Sagnik Nag, Sukhamoy Gorai, Nobendu Mukerjee, Dattatreya Mukherjee, Rishabh Vatsa, Mamtha Chandrakanth Jadhav, Arabinda Ghosh, Arikketh Devi, Anand Krishnan, Nanasaheb D. Thorat

**Affiliations:** † Department of Medical Biotechnology, Ramakrishna Mission Vivekananda Educational and Research Institute, Howrah, West Bengal 711202, India; ‡ Cancer and Stem Cell Biology Laboratory, Department of Genetic Engineering, SRM Institute of Science and Technology, Kattankulathur, Tamil Nadu 603203, India; § Centre for Nanosciences and Molecular Medicine, Amrita Vishwa Vidyapeetham, Kochi, Kerala 682041, India; ∥ Department of Biotechnology, School of Biosciences and Technology, Vellore Institute of Technology (VIT), Vellore, Tamil Nadu 632014, India; ⊥ Rush University Medical Center, 1620 W Harrison St, Chicago, Illinois 60612, United States; # Department of Microbiology, West Bengal State University, Kolkata, West Bengal 700126, India; g Department of Health Sciences, Novel Global Community Educational Foundation, https://www.ngcef.net/; h Raiganj Government Medical College and Hospital, Raiganj, West Bengal 733134, India; i Department of Microbiology, Vels Institute of Science, Technology and Advanced Studies, Pallavaram, Chennai 600117, Tamilnadu, India; j Jawaharlal Nehru Medical college, Wardha, Maharashtra 442001, India; k Microbiology Division, Department of Botany, Gauhati University, Guwahati, Assam 781014, India; l Department of Chemical Pathology, School of Pathology, Faculty of Health Sciences, University of the Free State, Bloemfontein, Free State 9300, South Africa; m Nuffield Department of Women’s and Reproductive Health, Division of Medical Sciences, John Radcliffe Hospital, University of Oxford, Oxford OX1 2JD, United Kingdom; n Department of Physics, Bernal Institute and Limerick Digital Cancer Research Centre (LDCRC) University of Limerick, Castletroy, Limerick V94T9PX, Ireland

**Keywords:** Eexosomes, cancer, drug loading methods, drug delivery, cancer biomarker

## Abstract

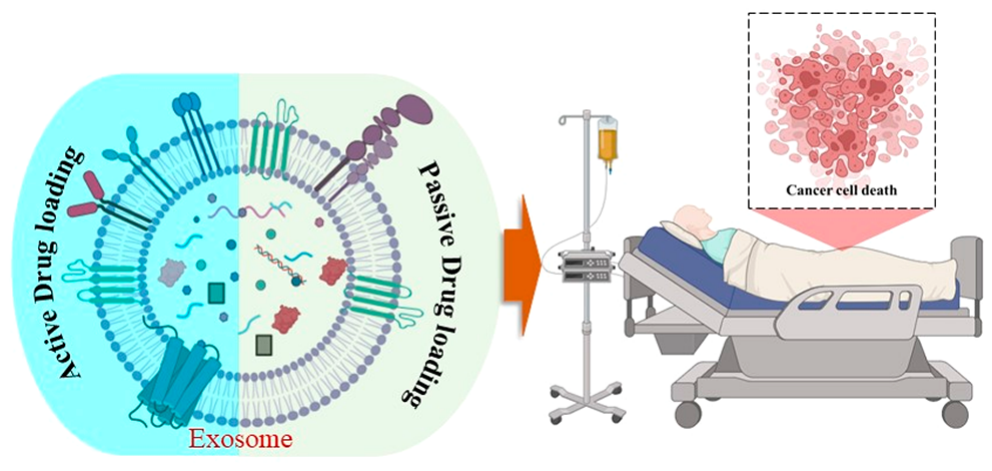

Exosomes are the phospholipid-membrane-bound subpopulation of extracellular vesicles derived from the plasma membrane. The main activity of exosomes is cellular communication. In cancer, exosomes play an important rolefrom two distinct perspectives, one related to carcinogenesis and the other as theragnostic and drug delivery tools. The outer phospholipid membrane of Exosome improves drug targeting efficiency. . Some of the vital features of exosomes such as biocompatibility, low toxicity, and low immunogenicity make it a more exciting drug delivery system. Exosome-based drug delivery is a new innovative approach to cancer treatment. Exosome-associated biomarker analysis heralded a new era of cancer diagnostics in a more specific way. This Review focuses on exosome biogenesis, sources, isolation, interrelationship with cancer and exosome-related cancer biomarkers, drug loading methods, exosome-based biomolecule delivery, advances and limitations of exosome-based drug delivery, and exosome-based drug delivery in clinical settings studies. The exosome-based understanding of cancer will change the diagnostic and therapeutic approach in the future.

## 1 Introduction

**Figure 1:**
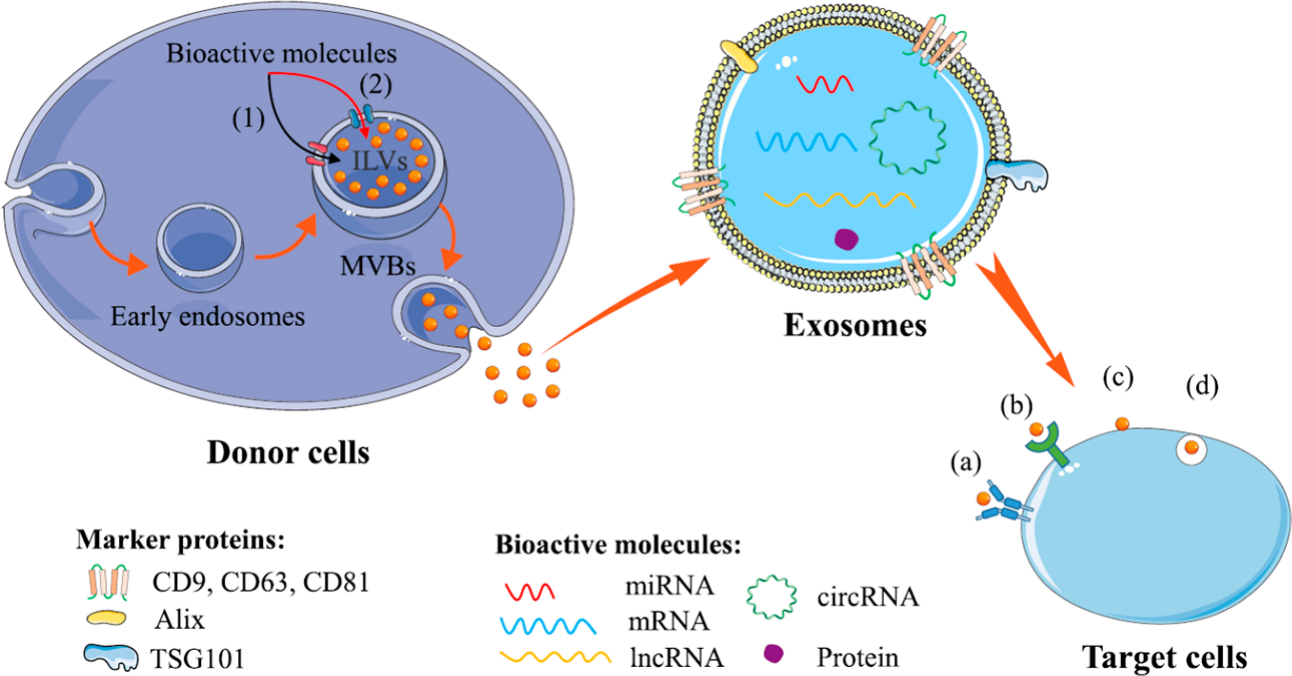
Overview of exosome biogenesis via (1) the ESCRT-dependent pathway and (2) the ESCRT-independent pathway involving exosome biogenesis and cargo selection of molecules. Target cells uptake exosomes via different pathways such as (a) antigen presentation, (b) cell signaling, (c) cell membrane fusion, and (d) pinocytosis or phagocytosis. Reproduced with permission from ref (12). Copyright 2020 Elsevier.

Exosomes are nanoscale extracellular vesicles secreted from several cells.^1,2^ This is the most fast-growing research field. The most interesting thing about the exosome is that it is the messenger of several pathological conditions. The fundamental level is involved in cellular communication.^3^ It transports several biologically active cargoes, for example, DNA,^4^ RNA,^2,5^ proteins,^6−8^ etc. This cargo can transform the cellular behavior of uptaking recipient cells. Cancer and exosomes have the most thrilling association. The collective evidence shows that tumor-derived exosomes (TEXs) regulate cell signaling and reprogramming in the complex tumor microenvironment (TME) to promote cancer development (uncontrolled cell growth, angiogenesis, metastasis, organ-specific metastasis immune evasion, and drug resistance).^9−11^ TEXs carry the molecular signature to help the early detection of cancer and work as biomarkers of cancer. Multiple nanodrug delivery technologies are being studied to improve medication potency, minimize toxicity, increase efficacy, and prolong drug flux duration. Early endosomes first develop when endocytic vesicles on the plasma membrane protrude outward. After changing into late endosomes, the early endosomes start to build up intraluminal vesicles (ILVs) in their lumen. This happens when the endocytic membrane enlarges inward. Endosomes ILVs are frequently referred to as MVBs due to their outward appearance. One group of bioactive molecules integrated into ILVs during MVB synthesis includes proteins, mRNA, miRNA, lncRNA, and circRNA (Figure 1^12^).

Exosome formation and biological cargo selection and loading are regulated via (1) the ESCRT-dependent process and (2) the ESCRT-independent pathway. ILVs are eventually discharged as exosomes into the extracellular environment when MVBs fuse with the plasma membrane. Several mechanisms, including (a) antigen presentation, (b) cell signaling, (c) cell membrane fusion, and (d) pinocytosis or phagocytosis, might lead to the uptake of these exosomes by target cells. In the drug delivery sector, natural or synthetic polymers and liposomes are more explored members. Both efficient drug delivery systems have several limitations, for example, low stability, toxicity, and low biocompatibility.^13^ In this crisis, exosomes show a promising role in drug delivery in in vivo and in vitro systems.^14^ Exosomes are overcoming all limitations of polymers and liposomes, which is the reason why they are becoming the brightest star in the drug delivery research area.^13,15^ In this review, we will cover exosome biogenesis, exosome sources, the exosome isolation process, the interrelation between exosomes and cancer, exosome drug loading methods, and the application of exosomes against several cancers and finally highlight the clinical study related to exosome-based drug delivery.

## 2 Biogenesis of Exosomes

**Figure 2:**
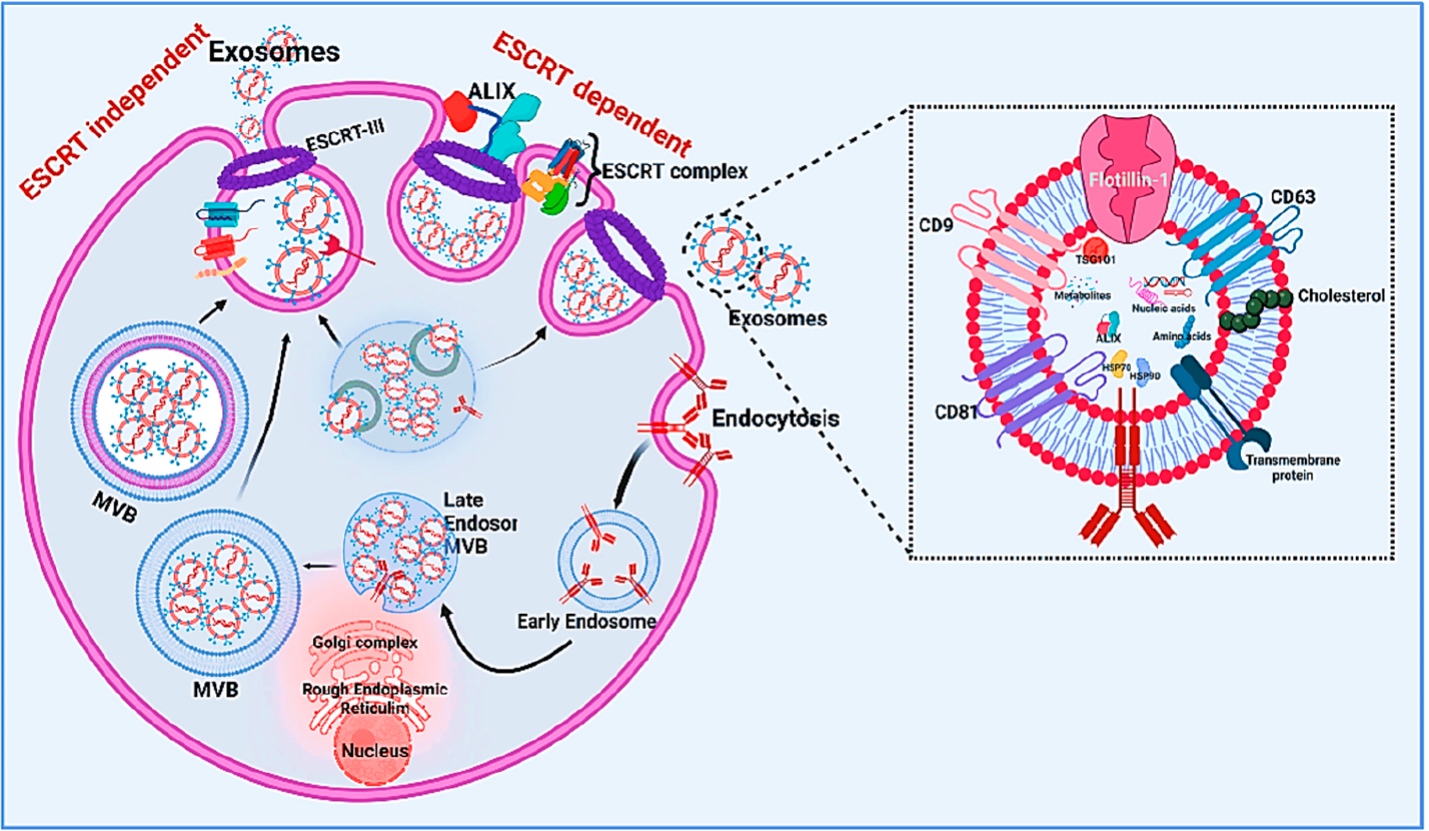
Biogenesis of an exosome and its components. This image explains the ESCRT-dependent pathway and the ESCRT-independent pathway of exosome biogenesis in a more detailed manner and explains exosome-related structure components. Created with BioRender.com.

Exosomes are dynamic entities continuously generated from the endosomal system within the cell and exposed to the extracellular environment through the process of exocytosis. The membrane of the multivesicular body (MVB) invaginates to form the late endosomal system, further elongating the late endosomes in the fold to form intraluminal vesicles (ILVs).^16^ During the formation of the ILVs, some specific proteins are incorporated into the vesicles, and these vesicles fuse with the perimeter or plasma membrane of the cell; these vesicles are termed exosomes.^17^ An interesting point about the structures of exosomes is that they are cup-shaped or biconcave when artificially produced by drying but in solution appear spherical when observed under the transmission electron microscope.^18^ There are many reports from the previous literature that some of the intricate protein machinery contributes to the formation of ILVs. This protein complex is termed the transport-required endosomal sorting complex, or ESCRT.^19^ Four different ESCRT subunits (0, I, II, and III) play key roles related to MVB formation, protein sorting, and cargo transport.^20^ ESCRT-0 binds to ubiquitinated protein-specific endosomal membrane domains with the help of its ubiquitin-binding domain and thereby initiates the ESCRT mechanism. After this initiation, ESCRT-0 interacts with ESCRT-I and then with ESCRT-II, and the whole complex then connects to ESCRT-III, which ultimately helps promote vesicle budding. Then, the splitting of the buds occurs. A specific sorting protein, Vps4, is present to provide the energy that separates the ESCRT-III complex from the MVB membranes. TSG101 and CHMP4 are also linked to the generation of exosomes. Budding and secretion to the extracellular membrane are regulated by EXCRT protein complexes.^21^ However, there are also pieces of literature demonstrating ESCRT-independent pathways for cargo sorting. In 2013, Airola et al.^22^ revealed that raft-based microdomains in the plasma membrane help in the lateral segregation of cargoes in the endosomal membrane. Interestingly, these rafts are highly enriched with sphingomyelinases, which are essential enzymes for the formation of ceramide through the hydrolysis of phosphocholine. In these ceramide-dependent pathways, the lateral phase separation is induced by ceramide and also promotes the spontaneous formation of cone curvatures in the plasma membrane, aiding the budding process.^23^ The biogenesis of the exosome pathway is explained in Figure 2.

Regardless of the regulation of biogenesis, sorting, and budding, one chromaticism of the exosome is that it is comparatively smaller and more uniform in shape. This makes exosomes able to escape mononuclear phagocytes, reducing their circulation time and increasing cell-to-cell communication.^24^

## 3 Fundamentals of the Exosomes

### 3.1 Structure and Composition of Exosomes

**Figure 3:**
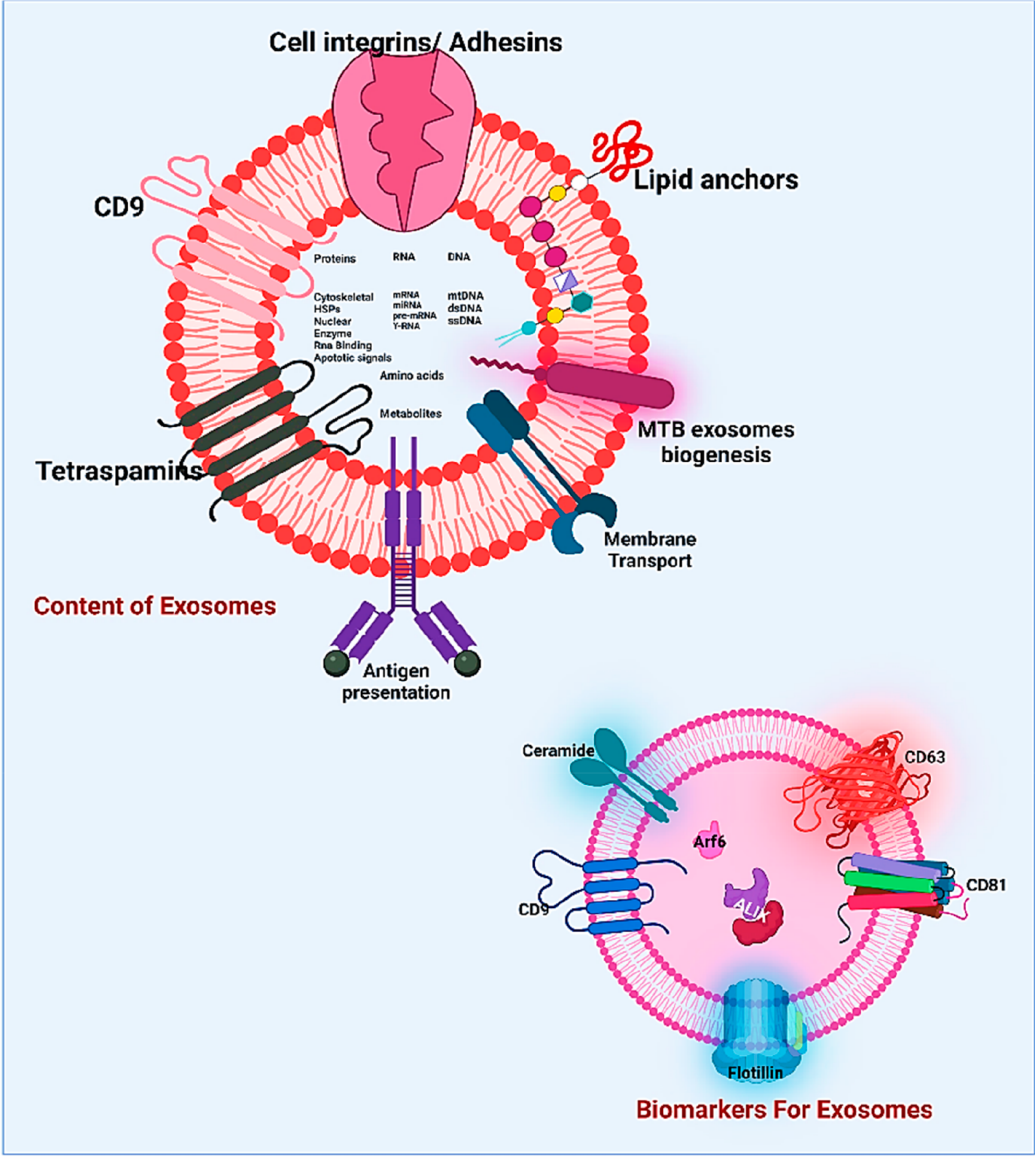
Structure and composition of exosomes. Multiple exosome-associated components (protein, DNA, RNA, and surface marker) play vital roles in cancer biomarkers. Created with BioRender.com.

Exosomes construct a phospholipid outer envelope, and the inner core carries a group of biologically active molecules.^3^ Components of exosomes are proteins, lipids, nucleic acids, and glycoconjugates (Figure 3).

Exosome surface proteins play a principal role in cellular communication (such as integrins and tetraspanins). Tetraspanins mainly regulate cell communication facilitated by exosomes, and CD9, CD63, and CD81 are mainly observed. Not only tetraspanins but also many adhesin proteins help exosomes fix with the recipient cells.^25^ There are also reports showing the involvement of integrins in exosome-mediated metastasis. In 2015, Hoshino et al.^26^ showed the horizontal transmission of α6β4 and α6β1 to the lungs and the horizontal transmission of αvβ5 to the liver, which ultimately promoted metastasis to the respective organs. The main source of lipids in exosomes is the plasma membrane of the parent cell from which the exosomes originate, but apart from the plasma membrane exosomes can also be produced from Golgi membranes.^27^ Exosome membranes contain multiple lipid molecules, including ceramide, cholesterol, phosphatidylcholine, phosphatidylserine, phosphatidylinositol, sphingomyelin, phosphatidylglycerol, and many more.^28^ They also carry dynamic nucleic acids (mRNA, circRNA, tRNA, piRNA, tRNA, sncRNA, rRNA, lncRNA, mtDNA, and dsDNA).^2,29−33^

### 3.2 Exosome Sources

Exosomes are isolated from various biological fluids (blood, urine, saliva, etc.).^34,35^ The other sources of exosomes are from the tumor microenvironment, since a large number of exosomes are produced in tumors compared to normal cells. Apart from that, exosomes can come from HEK293 cells (human embryonic kidney cells), HeLa cells, and many more. Aside from being a source of cancer, exosomes are also a source of other notable substances. DCs, NKs, and exosomes released from tumor cells are the major sources of vaccine development. There have also been reports of exosome-based cancer vaccines made primarily from mesenchymal stem cells and macrophages.^36,37^

### 3.3 Exosome Isolation

**Figure 4:**
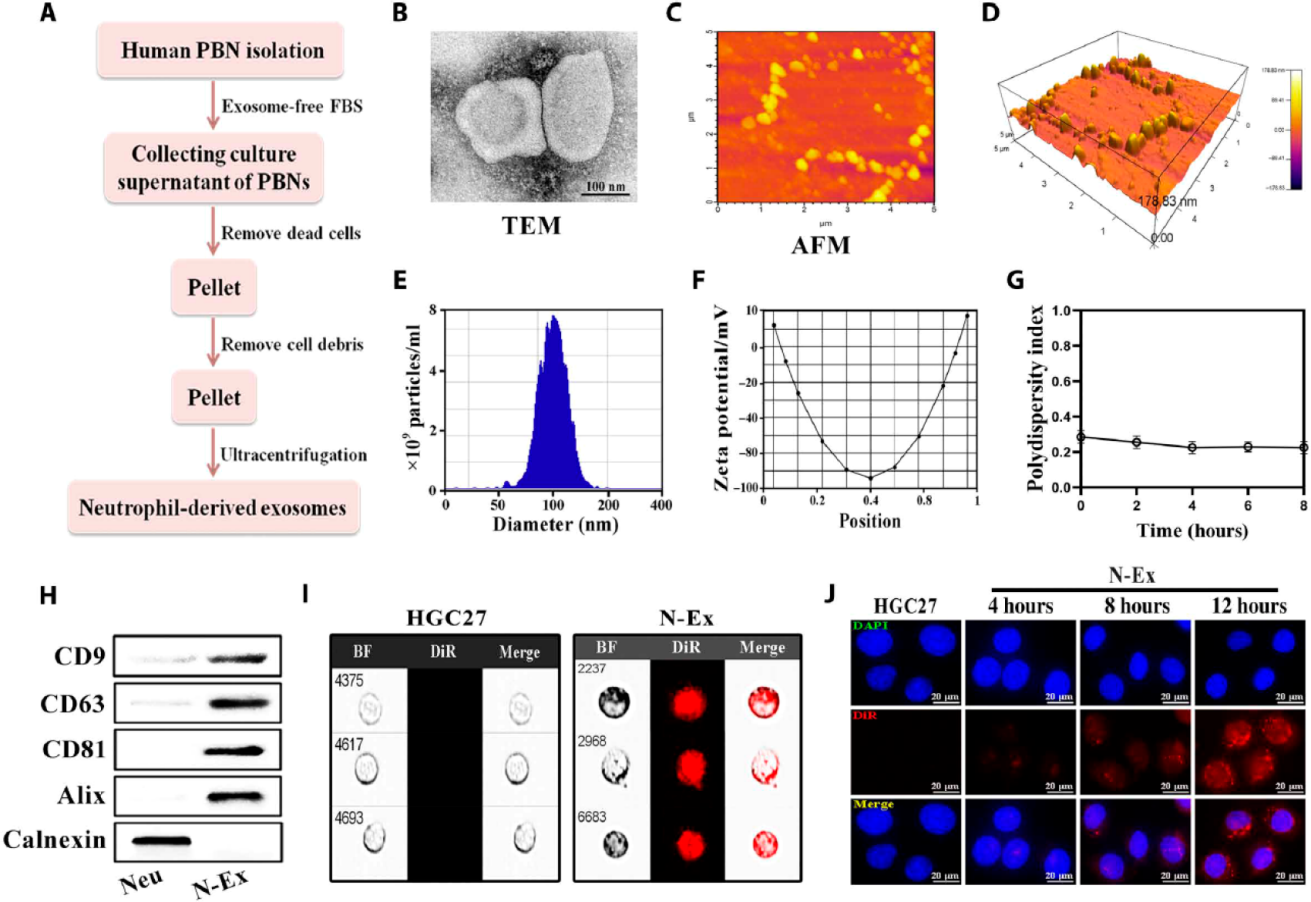
Neutrophil-derived exosome (N-Ex) isolation, characterization, and cell uptake. (A) Human peripheral blood neutrophil (PBN) isolation method. (B) Transamination electron microscopy analysis of N-Ex with 100 nm regulation. (C and D) Morphological analysis of N-Ex via Atom force microscopy (AFM). (E and F) Nanoparticle tracking assay (NTA) of N-Ex for size determination. (F and G) Explanation of the surface charge via ζ-potential analysis. (H) N-Ex surface marker analysis by Western blot with calnexin as the control. (I and J) Experimental analysis of DiR-labeled N-Ex untacking in the gastric cancer cell (HGC27) via (I) imaging flow cytometry and )J) fluorescence confocal laser microscopy. Cell nuclease staining was done by 4′,6-diamidino-2-phenylindole (DAPI) and bright field (BF) with 20 μm regulation. Reproduced with permission from ref (48). Copyright 2022 AAAS.

The isolation of exosomes is the most challenging process in EV research. There are several isolation methods, such as ultracentrifugation,^38^ density gradient centrifugation,^39^ ultrafiltration,^40^ size exclusion chromatography (SEC),^41^ immunoaffinity, and polymer precipitation.^39^ Each method has advantages and disadvantages. Some of the advanced techniques^42^ to isolate exosomes include microfluidics,^42,43^ lipid nanoprobes,^44,45^ and thermo-acoustofluidic separation.^46,47^ Exosome isolation is related to several methods, and their advantages and disadvantages are summarized in Table 1. In the experimental aspect, the appearance of an isolated exosome and exosome-specific biomarker analysis are explained in Figure 4.^48^

**Table 1:** Exosome Isolation Methods

isolation technique	mechanism	advantages	disadvantages	references
ultracentrifugation	components with varying sizes and densities have varying sediment speeds	a gold standard, ideal for large-scale samples, inexpensive, and the isolation procedure requires more than 4 h	exosomes may be damaged, the procedure is time-consuming and inconvenient, the purity is modest because of nonexosomal component contamination, and the yield is low	(38)
density gradient centrifugation	components with varying sizes and densities have varying sediment speeds	exosomal damage is avoided, hence we acquire high purity, and the procedure is completed in more than 16 h	preliminary preparation is labor-intensive, the procedure is time-consuming, and the yield is minimal	(39)
ultrafiltration	particles of varying sizes and molecular masses	it is simple and does not require any special equipment or reagents, it takes less than 4 h to complete, the component’s purity is high, and the yield is moderate	exosomes with tiny particle diameters are lost due to clogging on the filtering membrane	(40)
size-exclusion chromatography (SEC)	different sized and molecular density particles	exosome subtype isolation has a high level of specificity and it takes 0.3 h for qEV (Izon Science, New Zealand), which has a high yield and purity	lipoprotein contamination necessitates the use of special columns and packing	(41)
immunoaffinity	based on the interaction of antibodies with specific exosome membrane proteins	exosome subtype isolation has a high level of specificity, it takes between 4 and 20 h, has a high purity but limited yield	depending on the antibody’s specificity, it can be quite costly	(39)
polymer precipitation	the effect of exosomes on the solubility or dispersibility of high hydrophilic polymers	the simple technique takes between 0.3 and 12 h and is ideal for large-volume samples and the yield is high	contaminants may be present as a result of copurifying protein aggregates or residuary polymers, resulting in a low purity level.	(39)
microfluidics-based techniques	in this process, fluid runs through via a microchannel and captures the exosome based on a surface marker	this process supports isolated exosomes with high purity, reduced chemical utility and fast detection	expenses of this technique and it only applicable for small scale sample and have probability of losing exosome during washing time	(42, 43)
lipid nanoprobes	magnetic probe-mediated affinity-based exosome separation	its capable large-scale sample processing for protein and nucleic acids analysis	isolated exosome purity medium	(44, 45)
thermo-acoustofluidic separation	this process separated the exosome based on the lipid ratio	this process capable remove other extracellular vesicles contamination from exosome	protein contamination	(46, 47)

## 4 Exosomes for Cancer Theragnostic

### 4.1 Exosomes and Cancer

The association between exosomes and cancer is the most highlighted area of current research. The unexplained nature of exosomes has raised concerns about multiple events and their role in cancer cell angiogenesis and metastasis, including epithelial to mesenchymal transition (EMT) and immunological modulation.^49^ TEXs (tumor-derived exosomes) play an important role in the origin, development, and treatment resistance of cancer.^50,159^ The discovery of exosomes, which serve as regulatory agents in cancer intercellular communication, increases the potential to investigate the understanding of tumor immunity. Several scientific studies suggest that tumor-associated macrophages (TAMs) are involved in major inflammation, suggesting that TAMs play a significant role in tumorigenesis.^51^ According to various studies, TAMs promoted multiple cells in macrophage polarization.^52^ TAMs lose their anticancer activity and promote tumor progression. Exosomes released from the tumor reprogram the macrophages and support cancer development.^53^ Hypoxia is another important feature of the TME (tumor microenvironment) related to immunosuppression. Hypoxic conditions influenced the tumor-cell-derived exosome to drive cancer to a more aggressive pattern.^54^ The epithelial to mesenchymal transition (EMT) can be regulated by several transcription factors.^24^ Hypoxic tumor cells, derived from multiple molecules of exosomes, reprogram the immune system and promote cancer development. This exosome miRNA cargo affects macrophage function and M2 polarization.^55^ Exosomes are associated with multiple miRNAs associated with tumor progression.^56,57^ The exosome circular RNAs play a crucial role in the cellular communication that occurs in the tumor microenvironment. In addition to RNA, proteins also play a crucial role in tumor progression. Matrix metalloproteinases (MMPs) are related to cells with cellular adhesion properties, and TEXs alter MMP functions, causing cells to become motile. It was discovered that M2 macrophage-derived exosome CD11b/CD18, an integrin, promotes cancer cell proliferation while inhibiting metastasis by activating MMP-9. Due to its antiatherogenic effects, apolipoprotein E (ApoE) is an important protein molecule involved in M2 polarization. TAM releases IL-1, VEGF (vascular endothelial growth factor), and cytokines that participate in tumor development. Exosomes, which carry multiple cargoes to accelerate angiogenesis, were recently discovered to play a critical role in cancer invasiveness.^53^ In cancers, TEXs are also responsible for theepithelial to mesenchymal transition (EMT).^58^ The surface integrin of exosomes leads to organ-specific metastasis. TEXs-guided cancer cell migration in a specific organ is regulated via the diversity of TEX integration.^26^ The transcriptional regulator GATA3 was abundantly released from TAM-derived exosomes, where it plays an important role in epigenetic modulation to induce angiogenesis and EMT.^53^

### 4.2 Exosome is the Source of Cancer Biomarkers

The molecular contents of exosomes normally reflect those of their parent cells and can therefore be used as biomarkers for pathophysiological complications (such as cancer).^59,35,158^ Tumor and stromal cells in the TME have been reported to release exosomes, and their molecular signatures play a dynamic role in cancer.^60,61^ For example, it has been found that TNBC (triple-negative breast cancer) cells with CCL5 on their surfaces, derived from tumor-derived exosomes, alter TME-associated macrophages and develop a metastatic nature, resulting in a TME favorable for carcinogenesis.^62^ Researchers suggest that derived cancer stem cells are involved cancer metastesis.^63^ TEXs are being studied as diagnostic and prognostic biomarkers in clinical trials. A clinical study NCT04523389 related to colon cancer focuses on the development of diagnostic markers. TNBC TEVs carry multiple molecules that are sources of diagnostic and prognostic biomarkers.^60^ Some of the most complicated cancers, such as breast cancer,^64,69^ lung cancer,^65,70^ colon cancer,^66,71^ prostate cancers,^67,72^ and liver cancers,^68,73^ and their related exosome biomarkers with clinical importance are discussed in Table 2.

**Table 2:** Exosome-Associated Cancer Biomarkers and Their Clinical Significance

biomarker	cancer	source	exosome component	clinical significance	reference
diagnostic	breast cancer	plasma	miR-223-3p	early diagnostic breast metastasis biomarker	(64)
	lung cancer	serum	miR-106b	it is highly expressed in serum and it is also associated with lymph node metastasis and mmp protein expiration in lung cancer metastasis	(65)
	colon cancer	plasma	CD147	it highly expresses in colon cancer patients	(66)
	prostate cancers	urine exosome	miRNA-501-3p	it is downregulated in prostate cancers but suppresses E-cadherin expression and promotes metastasis	(67)
	liver cancers	serum	circRNA-100338	it enhances liver cancer metastasis	(68)
prognostic	breast cancer	plasma	miR-222	it is interlinked in breast cancer (highly expressed) with lymphatic metastasis	(69)
	lung cancer	plasma	miR-451a,	it participates in lymph node metastasis in lung cancer	(70)
	colon cancer	serum	miRNA-203	it highly expressed colon cancer and is associated with metastasis, in vivo model (liver metastasis)	(71)
	prostate cancers	plasma	miR-1290 and miR-375	it highly expressed prostate cancer and is related to castration-resistant poor overall survival	(72)
	liver cancers	serum	miR-1262	it is an efficient prognostic biomarker of liver cancer	(73)

### 4.3 Exosomes as Carriers

Exosomes are nanosized extracellular vesicles released by multiple cells. Exosomes with a wide size distribution are easier to internalize, as cells prefer smaller exosomes.^64^ Because of their economy of scale and immense potential in drug therapy, they have been an important research area in biomedicine and biomaterials.^65^ Exosomes are released into the surrounding body fluids. They have been shown to contain the molecular signatures of the parent cells (such as proteins, DNA, RNA, and lipids). This signature molecule acts as a messenger of cell status. Exosomes are the most interesting noninvasive diagnostic biomarkers and therapeutics. Their cargo molecules are involved in cellular communication.^24,60^ The secretion of exosomes from specific cells or tissues is based entirely on the cellular and philological condensation of cells.^66^ The exosome leads to biologically active molecules.^67^ The exosomal molecular signature has a complex association with multiple treatment resistance and carcinogenesis.^60^ miRNAs associated with TEXs promote EMT (miRNA-21, miRNA-92b, miRNA-130a, miR-149, miRNA-181c, miRNA-200, miRNA-328, miRNA-423-5p, miRNA-602, and miRNA-1246), tumorigenesis, invasion, and metastasis (let-7a miRNA, miRNA-21, miRNA-221/222, and miRNA-42.^68^

### 4.4 Routes of Administration

Understanding and comprehensively analyzing the underlying complexity of cellular communication is a potential tool for the development of efficient drug delivery systems and therapies in the fight against cancer. In the past decade, significant research in the field of exosomes has gained momentum. The whole situation regarding their cellular interactions with disease progression has yet to be fully explored.^69^ Recent scientific expeditions have documented effective exosome-mediated therapeutic delivery to cancer models and provided insights to improve disease pathophysiology.^70^ Efficient drug loading and sustained drug release via exosomes in and around the tumorigenic tissue depends on a complex, multifaceted set of factors.^71^ Based on clinical data and other medical research, there are specific drug delivery routes that exosomes should follow in order to reach the tumor target site.^71,42^ Nowadays, several conventional and unconventional routes of administration for these vesicles have been tried by several clinical research groups, namely, parenteral, oral, intertumoral, intranasal, and intraperitoneal routes.^72−74^ Needless to say, the appropriate choice of the route of administration of the drug in relation to the type of cancer it is dealing with is absolutely essential to the success of exosome delivery. Considering all the challenges and adversities, these exosome-based drug delivery targets pave a new way toward successful drug delivery and sustained drug release strategies in various tumors.^74^ Appropriate clinical trials and research need to be standardized to target potential exosomal agents to combat the growing rates of cancer.^75,76^

### 4.5 Exosome Loading Method

**Figure 5:**
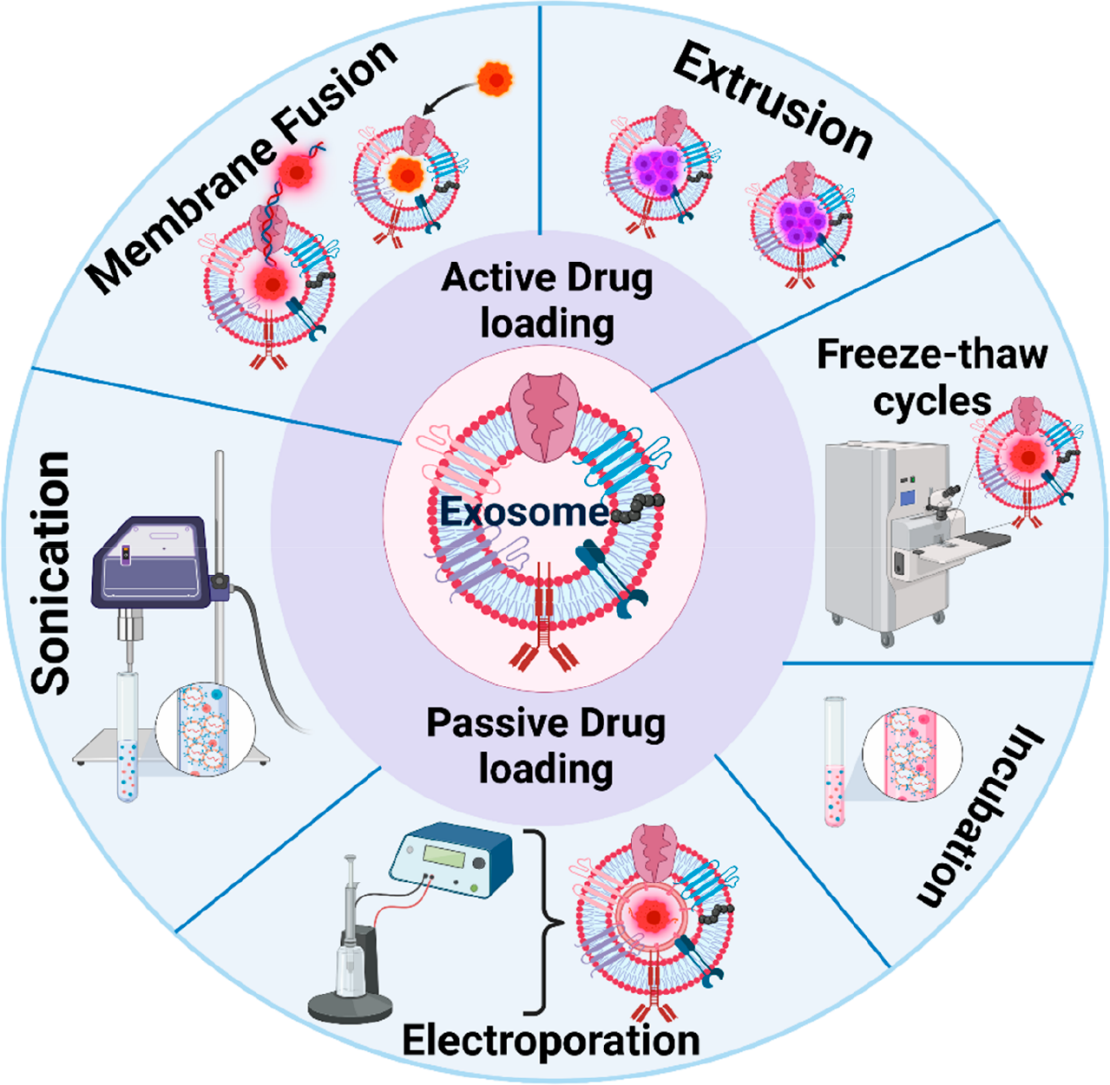
Exosome drug loading methods. Exosome drug loading methods are classified into two major classes: active (electroporation, sonication, fusion method, freeze–thaw cycles, and use with membrane permeabilizers) and passive (incubation). Created with BioRender.com.

Exosomes are natural carriers into which drugs can be loaded. Exosomes are encapsulated with drugs to make them suitable for the various target therapies. There are three different methods by which drug encapsulation occurs: the postloading method, the preloading method, and the fusion method.^77^ Therefore, since the incorporation of the drug into this lipid bilayer membrane is challenging,^78^ two different methods are followed; active loading and passive methods^79^ (Figure 5).

In active or remote or postdrug loading, the cells are cleaned to obtain a naïve exosome that is then sealed with drugs, while in passive loading or preloading methods the cells and the drugs are incubated together and the component later undergoes purification to yield a drug-sealed exosome. The postloading method works better with hydrophobic drug components than hydrophilic drug components.^80^

## 5 Active Drug Loading Approaches

Rupture of the exosome membrane is used to allow the entry of functional components into the exosome during drug loading. After the required molecules are loaded into the exosomes, the exosome retains its previous shape. Electroporation sonication, extrusion, and freeze–thaw cycling are some of the methods used to disrupt exosome membranes.^77^ Studies suggest that the active drug loading method increases the drug encapsulation efficiency of exosome development 11-fold.^81^ The limitation of this method is that it can affect exosome targeting properties and the native structure during the membrane rupture process.^77^

### 5.1 Electroporation

Electroporation involves a high-intensity electric field, instantaneous changes in cell membrane permeability, and drug loading. The voltage settings for different types of donor cells, such as B. Hela cells, monocytes, and immature dendritic cells, generally range from 150 to 700 V.^77^ Drug molecules enter through holes created in the exosome membrane during electroporation, while the membrane is restored after loading. This approach is commonly used to load large molecules such as miRNAs and siRNAs^81^ into exosomes. The electroporation process has a poor loading capacity due to the aggregation of RNA and stability issues. This approach can improve the loading of hydrophilic small molecules in exosomes and increase the efficiency of RNAs in exosomes.^81^

### 5.2 Sonication

The premise of ultrasonic drug loading is that ultrasonic waves lower the microviscosity of the membrane (usually by at least twofold), allowing the hydrophobic drug to pass.^77^ Exosomes derived from parental cells or recipient cells are mixed with a specific drug and protein legend before being sonicated with a homogenizer probe. The integrity of the exosome membrane is disrupted by the mechanical shear stress generated during sonication, allowing bioactive chemicals to enter the exosome while the membrane is deformed.^80^ Research suggests that sonication alters the viscosity of exosomes,^68^ but there are no reports of a reduction in the membrane-bound protein or lipid content of the exosome.^80^ After a 1 h incubation at 37 °C, it was shown that the membrane integrity of the exosome was restored. Drugs that bind to the surfaces of exosomes release very quickly, and drugs encapsulated via the exosome take time to release phage.^82^

### 5.3 Fusion Method

Membrane fusion, itself a scientific achievement, can fuse exosomes and nanocomposites within a membrane structure. It allows for the prolonged release of nanodrugs, enhances absorption and efficacy, and performs an exocrine function in immune system response, antigen presentation, cell migration, cell differentiation, and tumor invasion.^77^ This adaptable technique was successful in enriching exosomes using hydrophilic biological components without removing their function. When a drug and a liposome-encapsulated drug were compared, hybrid EVs increased the cellular transport efficiency of a chemotherapeutic agent by three- to fourfold. Fluorescence resonance energy transfer, which detects changes in nanoscale spacing of biological macromolecules in vivo, was used to confirm the hybrids.^83^

### 5.4 Freeze–thaw Cycles

Exosomes are incubated with selected drugs at room temperature for a set period before being quickly frozen at −80 °C or in liquid nitrogen. Thereafter, the combination is allowed to thaw at room temperature. Freeze–thaw cycles are performed at least three times to improve drug encapsulation. Compared to sonication or extrusion, this method has a reduced drug loading capacity. Furthermore, this approach can increase exosome aggregation, resulting in large-scale drug loading of exosomes.^84,85,83^

### 5.5 Used with Membrane Permeabilizers

Membrane permeabilizers and surfactants such as saponin can interact with cholesterol in the cell membrane to create pores that allow the passage of exosomes. The membrane permeability approach can improve the loading efficiency of catalase into exosomes compared to the incubation method.^80^

## 6 Passive Loading Approach

The method involves the integration of drugs with exosomes. The mechanism of encapsulation and its loading efficiency depend on the hydrophobic interaction and diffusion between the loaded molecule and the lipid layer of the exosomes.^85,77^

### 6.1 Incubation

The passive loading approach involves two different types of incubation: incubation of drug along with an exosome or with donor cells. In the case of incubating a drug with exosomes, this technique allows the drug to enter the exosome based on the concentration gradient during the incubation. Since hydrophobic drugs can interact with the lipid surfaces of exosomes, this property is exploited for drug loading.^80^ In one study, exosomes were incubated with the paclitaxel stock solution for 1 h at 22 °C to produce an excipient preparation with a loading efficiency of 9.2%. Based on the high lipophilicity and limited water solubility of paclitaxel, this technique uses the passive diffusion of drugs packaged in exosomes. In addition, it has been suggested that coincubation at 37 °C can be used to load miRNAs into exosomes.^77^ The disadvantages of this method is that it is limited to a specific type of drug and the amount released after incubation is not sufficient for clinical trials.^86^ In the case of incubation with donor cells, the drug is coincubated with the donor cells, which is done by pretreating the cell membrane, and then exosomes loaded with the drug are shed using UV light, heat, or both. In both cases, the cell membrane is unobstructed, but the downside that researchers face during incubation is the insufficient number of exosomes that are secreted.^13^ The efficiency during loading and the cytotoxicity that cells experience while responding to the drug also pose research challenges.^80^

### 6.2 Drug Delivery via Exosomes

The latest discoveries point to a unique property of exosomes, as it was found that exosomes can transport proteins and genetic and epigenetic information from one cell to another cell through receptor–ligand interactions.^87,88^ One of the results suggests that exosomes obtained from mouse mastocytes can be transferred to humans and the RNA obtained from this transfer can be used in other humans and mice.^2^ Discoveries stated that exosomes self-decode upon transfer into recipient cells according to the host body, hence protein translation in the host body occurs depending on the host physiology.^89^ The uniqueness of the exosome makes it the most important medium for transporting drugs to the cells.^48^ Unlike other carriers used in cells, such as liposomes and polymeric nanoparticles, exosomes have the unique potential of being an endogenous cellular machinery that can be used for drug delivery and storage.^90^ Exosome delivery enables simultaneous intercellular communication by sending many signals simultaneously. Exosomes are unlikely to be freely circulating soluble factors and can release large amounts of functional molecules, as they are soluble factors in the host cells.^91^ Exosomes have other additional properties such as the protection of the protein or drug entrapped within due to their small size, which helps exosomes avoid phagocytosis.^13^ Exosome cargoes can travel long distances, have high biocompatibility, are nonimmunogenic and targeted, and can overcome a variety of physical barriers due to their properties.^13,92^

### 6.3 Delivering Small Molecules via Exosomes

Drugs can be encapsulated in exosomes, thereby prolonging the drug half-life and improving the stability of drug release. Furthermore, due to their endogenous origin, exosomes are highly biocompatible and can be used as nanocarriers for tissue-specific targeted delivery.^86^ Studies show that exosomes were designed with hydrophobic agents such as curcumin, and the results showed that exosomes could carry the hydrophobic agent and also enhanced its anti-inflammatory properties.^93^ Various studies conducted have found that exosomes can cross the blood–brain barrier. This scientific evidence suggests that exosomes overcome nanoparticles based on multiple membrane cross-constraint. This attribute of the exosome makes it a more efficient drug delivery tool.^94^ From this we can conclude that exosomes can not only transport the drugs but also increase their half-life, reduce toxicity, and even overcome various barriers.

#### 6.3.1 Delivering Proteins via Exosomes

Exosomes are also used to carry large molecules, such as proteins, in addition to tiny compounds. To understand the role and importance of exosomes in protein delivery, we can consider a case related to Parkinson’s disease (PD).^13^ Exosomes produced by the central nervous system (CNS) have been found in cerebrospinal fluid and peripheral body fluids, and several studies suggest that their molecular signatures play a role as biomarkers in Parkinson’s disease (PD). Exosomes have been shown to spread toxic α-synuclein protein (syn) between cells and cause apoptosis, suggesting a critical mechanism that causes the disease. This accelerates syn-aggregate proliferation in brain pathogenesis in Parkinson’s disease. However, exosomes have also been reported to play a significant role in the treatment of PD. In the mouse model of PD, researchers have found that exosomes transport catalase and small interfering RNAs to the brain.^95^ Designing exosomes with catalase can be said to be a promising therapy for PD therapy because the delivery of catalase across the BBB, like many other drugs, is challenging and exosomes have overcome this hurdle. The targeted delivery of armed exosomes is also used as an anticancer treatment, with the exosomes loaded with various active pharmaceutical ingredients (API), including genetic material, proteins, and chemotherapeutic agents.^96^ The exosomes have a more efficient ability to load anticancer drugs onto their surfaces compared to synthetic nanoparticles.

#### 6.3.2 Delivering Genetic Material via Exosomes

Various studies conducted have found that exosomes can carry both large and small molecules. These cargoes can be engineered to even carry genetic and epigenetic material.^87^ Gene therapy is being considered for the treatment of various types of cancer. Exosome-based gene therapy transports siRNA, mRNA, and miRNA along with exosomes.^97^ Exosomes are the most efficient miRNAs transporter tools and are used for therapeutic RNA delivery.^98^ Several studies show that exosomes transport RNA more efficiently than any other nanoparticle. Exosome-based small RNA delivery enhances its functional efficiency. Studies have shown that exosomal miRNAs molecules have a complex interrelationship in multiple cancer delivery phages (angiogenesis and metastasis).^97^

## 7 Application of Exosome-Based Drug Delivery in Multiple Cancers

Exosomes are nanosized extracellular vesicles. They secretes from almost all cells. The main contribution of exomes is in cellular communication.^99,100^ They have been found in body fluids such as blood, urine, cerebrospinal fluid, saliva, etc. This evidence proved that they are involved in several physiological metabolic processes.^100^ However, exosomes have also been shown to be involved in cancer development, progression, and metastasis. Tumor-derived exosomes (TDXs) have been reported to promote cancer proliferation and cause the formation of the premetastatic niche. They have also been found to regulate drug resistance.^13,100^ TDXs turn the recipient cells into cancer cells. Evidence has shown their involvement in the modulation of immune response, stromal cell reprogramming, extracellular matrix remodelling, the induction of drug resistance, etc.^101^ Exosome-associated molecular signatures are promising evidence for the invention of cancer biomarkers.^26^ The exosome-based therapeutic approach is the most innovative area in cancer research.^13,100^ This Review aims to summarize the clinical therapeutic exosomes that behave as nanocarriers that deliver nucleic acids, mRNAs, microRNAs, proteins, lipids, and metabolites to other cellular habitats and behave as convenient drug delivery systems.^25^ The exosomes are isolated from the patients and conjugated with drugs, and this approach develops biocompatibility and low toxicity in drug delivery.^102^ This system also bypasses the P-glycoprotein drug efflux system, thus reducing the risk of drug resistance.^82^ It has been reported by a research group that the exosome penetrates deep into the tissue, effectively diffuses in the blood, and even crosses the biological barrier.^103^ Exosomes can also be effectively engineered for cell and tissue specificity, allowing the increase of the drug concentration at a given diseased site.^104^ The potential applications of exosome-based cancer therapy are presented in Table 3. Homeostasis in a normal cell is maintained by the transfer of bioactive molecules across membranes. This diffusion and uptake of biological materials occurs through extracellular vesicles, which characterize the cargo and send it to its assigned destination. Exosomes are extracellular vesicles that moderate this intercellular communication. Previous studies have shown that exosome cargoes can hijack the cells in several pathological conditions such as cancer.^13,100^ Therefore, they have emerged as the essential regulatory molecules that modulate cell-to-cell communication during phage. The exosome has been shown to have an important interaction between tumor chemotherapeutic resistance and cancer metastasis.^105^ In the recent past, therefore, exosomes have been considered as important diagnostic biomarker sources and therapeutic tools against cancer. Although exosomes have shown promising results in vitro and in vivo, their use in humans as cancer therapeutics is still under investigation. Exosomes require more detailed study and understanding to become potential drug delivery systems and anticancer therapies in the near future.

**Table 3:** Exosomes for Targeted Drug Delivery in Cancer Therapy

therapeutic cargo	targeting ligand	target cell	function	method of synthesis	types of modification	reference
KRAS siRNA (Kirsten Ras oncogene short interfering RNA)	iRGD peptide (Arg-Gly-Asp peptide)	adenocarcinoma, human alveolar basal epithelial cells	targets oncogenic KRAS (Kirsten Ras oncogene)	LAMP-2B (Lysosome-associated membrane protein 2 gene)	genetically modified	(105)
DOX (doxirubin)	αv-integrin-specific iRGD peptide	breast cancer	targeted delivery of DOX (doxirubin)	LAMP-2B	genetically modified	(105)
SOX2 siRNA (silencing RNA)	tLyp-1 (linear truncated form of LyP-1)	nonsmall cell lung cancer, A549 stem cells	Gene delivery for cancer therapy	LAMP-2B	genetically modified	(106)
imatinib, BCR-ABL siRNA	IL-3	chronic myelogenous leukemia cells	inhibits cancer cell growth, increased intratumoral accumulation	LAMP-2B	genetically modified	(107)
5-fluorouracil anti-miRNA-21	zHER affibody	colorectal cancer	Reverses chemoresistance and improves cancer treatment efficiency	LAMP-2B	genetically modified	(108)
Tpd50 siRNA	DARPin	HER2-positive cells	RNAi therapy of HER2-positive cancer	LAMP-2B	genetically modified	(109)
miRNA-let7a	GE11 peptide	breast cancer	targets EGFR-expressing tumors	LAMP-2B	genetically modified	(110)
Smart-exos	αCD3/αEGFR	T cells (Jurkat), EGFR-positive breast cancer	cell-free cancer immunotherapy	Smart-exos	genetically modified	(111)
miRNA-26a	ApoA-1	hepatocellular carcinoma (HepG2)	suppresses tumor cell migration and proliferation	CD63	genetically modified	(112)
antigen	OVA antigen	CD8+ T cells	improves the immunogenicity of cancer vaccines	CD63	genetically modified	(113)(114),
Sstreptavidin-HRP, mannosamine	l-azidohomoalanine (AHA) (azide-bearing amino acids) and saccharides	biotin receptors	Florescence of cancer cells	Exosome azide integration, DBCO-PEG4-biotin–avidin conjugation	chemically modified	(115)
curcumin-SPION	neuropilin-1-targeted peptide	glioma	simultaneous diagnosis and treatment of glioma	click chemistry	chemically modified	(116)
paclitaxel (PTX)	AA	murine lung cancer, sigma receptor-positive cells	improves drug circulation and inhibits pulmonary metastases	DSPE-PEG-AA	chemically modified	(117)
quantum dot photothermal agent	RGD	breast cancer	near-infrared-II region quantum dot delivery for nucleus-targeted low-temperature photothermal therapy	DSPE-PEG-RGD	chemically modified	(118)
elastin	folate	breast cancer	targeted induction of ferroptosis	DSPE-PEG-folate	chemically modified	(119)
surviving siRNA	PSMA RNA aptamer, EGFR RNA aptamer, folate	breast cancer, prostate cancer, colorectal cancer	tumor-targeted RNAi nanomedicine	chol	chemically modified	(119)
miRNA-let7, VEGF siRNA	AS1411 aptamer	nucleolin-positive cancer cells	tumor-targeted small RNA delivery	chol	chemically modified	(119)
DOX	sgc8 aptamer	leukemia cells	targeted anticancer therapy	diacyl lipid-(PEG)2	chemically modified	(120)
PTX	AS1411 (aptamer-conjugated)	breast cancer	targeted anticancer chemotherapy	chol-PEG2000	chemically modified	(121)
methotrexate, KLA (Lys-Leu-Ala)	ApoA-1 mimetic peptide	glioma	selective brain tumor treatment (glioblastoma multiforme)	lipid	chemically modified	(122)
photosensitizer	NLS peptide	carcinoma (4T1), colorectal cancer (CT26)	dual-stage light-guided plasma membrane and nucleus-targeted photodynamic therapy	C16	chemically modified	(123)
aSIRPα, aCD47	antibodies	macrophages and tumor cells	enhance phagocytosis of cancer cells by blocking SIRPα-CD47 interaction	azide-modified	chemically modified	(124)
mannosamine	RGD	αvβ3 -overexpressing cells (HUVEC)	promote angiogenesis with targeted imaging	DSPE-PEG-RGD	chemically modified	(125)
SIRPα	mRNA	embryonic fibroblasts (MEFs)	increased exosome circulation time	CD47 surface decoration	chemically modified	(126)
copper-64 (64Cu)-radiolabeled polyethylene glycol (PEG)	N/A	diagnosis of cancer (passive action)	reduced exosome clearance enhanced tumor penetration	surface PEGylation	chemically modified	(127)

## 8 Clinical Applications of Exosomes in the Treatment of Cancer

### 8.1 Advancements and Limitations

Exosomes have great potential as new drug delivery vehicles due to their inherent involvement in intercellular exchange of biomolecules, particularly for biotherapeutics that can be loaded into exosomes using the cellular EV packaging machinery.^90^ As we discussed earlier, delivering a drug to target sites and crossing the barriers was possible through exosomes compared to other nanoparticles. The research data reported that the drug potency and half-life of exosomes were well maintained when they were introduced into the recipient cell. Since the exosome is a natural mediator, it has the natural ability of cell permeability, which helps it cross physical barriers and even escape lysosomal degradation and the endosomal pathway.^13^ Macrophage-derived genetically engineered exosomes are capable of drug delivery without rejection.^128^

There are several underlying questions that remain unanswered that limit the use of this novel component.^129^ (1) Industrial-scale production of exosomes would help treat cancer. (2) The storage of these exosomes derived from different cells and their longevity when not in use. (3) Targeting the armed exosomes to perform biogenesis at the site and not with other exosomes already present in the recipient cells. (4) The pathways and mechanisms that control exosomes will eventually help researchers fully control drug-containing exosomes. (5) One way to prevent therapeutic exosomes from reacting with healthy cells is to evaluate the characteristics of pharmacokinetics and pharmacodynamics, as well as safety, feasibility, toxicity, and pharmacodynamics. (6) Although exosomes are the natural mediator of cells, the immune response of a loaded exosome in the body has yet to be discovered.^13^ (7) We lack a technique that can help us to isolate exosomes with high purity and in reasonable quantities, which could help us to reduce costs, since exosome isolation is very expensive.^13^ (8) Hybrid exosomes are being used based on future demand, but the chemical efficacy and safety of such exosomes have yet to be investigated.^13^ (9) Exosomes are composed of heterogeneous components and have been reported to play an important role in tumor growth and even metastasis. Therefore, the immunogenic response of the hybrid exosomes or exosomes derived from other animals must be thoroughly investigated before they are used for clinical trials.^13^ (10) Although experiments show promising results in removing components from macrophage-derived exosomes by hypotonic treatment,^128^ the effect of the same treatment on exosomes bearing caspase-3 or other carcinogenic components remains to be investigated.^39^ (11) Most anticancer exosome drugs are still in the early stages of development.^130^

### 8.2 Exosome-Based Drug Delivery-Associated Clinical Trial for Cancer

Exosome-based clinical trials related to drug delivery are the most highlighted research area today. Sometimes they use a combination of traditional cancer therapy to develop effectiveness. Multiple cancer types and associated clinical trials of exosome-based drug delivery are constructively summarized in Table 4.

**Table 4:** Clinical Trials of Exosome-Associated Drug Delivery in Multiple Cancers

cancer type	drug used	clinical trial ID	sponsor	reference
breast cancer, Her-2 positive	trastuzumab emtansine	NCT01772472	funded by F. Hoffmann-La Roche/Genentech, KATHERINE ClinicalTrials.gov number NCT01772472.	(131)
breast cancer, triple-negative	atezolizumab with chemotherapy	NCT02425891	F. Hoffmann-La Roche/Genentech, IMpassion130 ClinicalTrials.gov number NCT02425891	(132)
breast cancer, Her-2 negative	ribociclib with endocrine therapy	NCT02278120	Novartis, MONALEESA-7 ClinicalTrials.gov number NCT02278120.	(133)
lung cancer	immunotherapy atezolizumab in combination with chemotherapy	NCT02763579	F. Hoffmann-La Roche/Genentech, IMpower133 ClinicalTrials.gov number NCT02763579.	(134)
colorectal cancer	encorafenib (BRAF inhibitor) plus cetuximab or encorafenib plus cetuximab and binimetinib	NCT02928224	Amgen (Inst), Bayer (Inst), Boehringer Ingelheim (Inst), Eli Lilly (Inst), Novartis (Inst), Roche (Inst), Celgene (Inst), Ipsen (Inst), Merck (Inst), Merck KGaA (Inst), Servier (Inst), Bristol-Myers Squibb (Inst). Genentech (Inst), Bayer (Inst), Pfizer (Inst), Eisai (Inst), Eli Lilly (Inst), Boston Biomedical (Inst), Daiichi Sankyo (Inst), Array BioPharma (Inst), Array BioPharma, GlaxoSmithKline, Novartis, Merck Serono	(135)
prostate cancer	combination of enzalutamide with androgen suppressor	NCT02446405	Astellas Scientific and Medical Affairs and others; ENZAMET (ANZUP 1304) ANZCTR number ACTRN12614000110684; ClinicalTrials.gov number NCT02446405, and EU Clinical Trials Register number 2014-003190-42	(136)
renal cell carcinoma	axitinib and pembrolizumab	NCT02853331	Merck Sharp and Dohme, KEYNOTE-426 ClinicalTrials.gov number NCT02853331.	(137), (138)
	combination of avelumab and axitinib	NCT02684006	Pfizer and Merck (Darmstadt, Germany), JAVELIN Renal 101 ClinicalTrials.gov number NCT02684006	
brain cancer	temozolomide with radiation selumetinib	NCT01149109	German Federal Ministry of Education and Research	(139), (140)
		NCT01089101	National Cancer Institute Cancer Therapy Evaluation Program, the American Lebanese Syrian Associated Charities, AstraZeneca	
lymphoma	rituximab with or without lenalidomide	NCT01938001	Celgene Corporation (Summit, NJ)	(141)
leukemia	c-methotrexate or high-dose methotrexate	NCT00408005	National Cancer Institute (NCI)	(142)
hepatoblastoma	minimal adjuvant chemotherapy	NCT00980460	National Institutes of Health.	(143)
melanoma	dabrafenib and trametinib	NCT01972347	GlaxoSmithKline; Novartis; National Health and Medical Research Council, Australia; Melanoma Institute, Australia.	(144), (145)
	ipilimumab and nivolumab	NCT02977052	Bristol-Myers Squibb	

## 9 Future Perspectives

Despite promising experimental achievements, there are some challenges in exosome-based drug delivery in terms of heterogeneity in origin, structure, and function. Among all these limitations, the greatest concern is the nonspecificity of exosome biodistribution. They can be found in various bodily fluids in the human body.^146^ However, in a study on BALB/c nude mice, it was observed that in the case of pancreatic cancer exosomes secreted by Panc-1 cells accumulate at the site of the tumor in a time-dependent manner. The rate of exosome accumulation is 30× higher than that of PEG–PE micelles at 4 h postinjection.^147^ Another major problem of exosomes is their ability to be rapidly cleared from the bloodstream after in vivo administration.^148^ This property is mysterious, since the exosome itself is made up of unique protein–lipid assemblies. However, the mystery was solved in a study that found the rapid clearance of exosomes from the bloodstream is due to uptake by macrophages. Experimental results clearly showed that exosomes derived from B16–B16 cells are quickly cleared after intravenous injection because liver and spleen macrophages have captured them.^149^ This problem can be solved to some extent by incorporating polyethylene glycol (PEG) into the structures of exosomes. It has been experimentally confirmed that exosomes with PEG can be detected even after 60 min postinjection, while exosomes without PEG can only be detected for 10 min.^150^ The implication of exosomes as drug carriers for unconventional therapeutics,^151^ including ocular, pulmonary,^152^ cutaneous, etc., is also difficult. To improve this, many parameters came into play. Two of the most important things are the penetrating power of exosomes in different tissues, tight junctions, etc. and their ability to evade the attack of tissue-resident immune cells and enzymes.^153^ The low yield of exosomes is a concern, as less than 1 g of protein is produced per ml of cell culture.^154^ Therefore, in order to conduct an experiment or clinical study, a large number of cells must be cultured. This limitation can be managed using exosome-mimetic nanovesicles.^155^ Exosome-mimetic nanovesicles (EMNV) can be produced by the serial filtration of extruded cells.^154^ It is reported that in this way the yield can be increased up to 100-fold.^155^ Plant-derived exosomes are some of the most frequent directions for research in the future. It has been reported that there are some exosome-related nanoparticles called folic acid-modified ginger-derived nanovectors that show very high compatibility and high potency while targeting cancer cells.^156^ In the case of FDA-approved nanomedicine research, the primacy of the exosome is limited. There are several aspects, including selecting the source of exosomes, standardizing techniques for culturing cells that produce exosomes, and isolating and quality controlling produced exosomes so that they can be applied to health-related problems, with particular reference to cancer. New technologies and regulations could reduce the boundaries of these fields.^153^ Finally, exosome-based research requires interdisciplinary^155,156^ work ecosystems that can develop an exosome-based advance therapeutic tool (such as a cancer vaccine^157^) for future cancer-associated global health problems.

## 10 Conclusions

Exosomes are burgeoning as next-generation platforms for nanomedicine in cancer therapy. It is clear that exosomes are used as promising biomarkers for several potential cancer types and also as an early detection tool in many clinical studies, some of which have already been discussed in this Review. The biocompatibility of exosomes and their highly specific interactions in living systems have stimulated the development of futuristic exosome-based therapeutic and drug delivery approaches. Genetically engineered exosomes loaded with specific drugs that target specific cancer cells offer more benefits compared to traditional cancer therapies. Nonetheless, this innovative approach also has some limitations in terms of difficulties in its scalability, purity, and isolation methods. This is the area where deeper research is needed. In the future, more efforts and more investigations will contribute to the development of this field, which will definitely open a new door through which we can be one step ahead of personalized medicine to treat cancer.

## References

[ref1] Harding C. V.; Heuser J. E.; Stahl P. D. Exosomes: looking back three decades and into the future. J. Cell Biol. 2013, 200 (4), 367–71. 10.1083/jcb.201212113. 23420870 PMC3575527

[ref2] Valadi H.; Ekstrom K.; Bossios A.; Sjostrand M.; Lee J. J.; Lotvall J. O. Exosome-mediated transfer of mRNAs and microRNAs is a novel mechanism of genetic exchange between cells. Nat. Cell Biol. 2007, 9 (6), 654–9. 10.1038/ncb1596. 17486113

[ref3] Raposo G.; Stoorvogel W. Extracellular vesicles: exosomes, microvesicles, and friends. J. Cell Biol. 2013, 200 (4), 373–83. 10.1083/jcb.201211138. 23420871 PMC3575529

[ref4] Balaj L.; Lessard R.; Dai L.; Cho Y. J.; Pomeroy S. L.; Breakefield X. O.; Skog J. Tumour microvesicles contain retrotransposon elements and amplified oncogene sequences. Nat. Commun. 2011, 2, 180 10.1038/ncomms1180. 21285958 PMC3040683

[ref5] Skog J.; Würdinger T.; van Rijn S.; Meijer D. H.; Gainche L.; Curry W. T.; Carter B. S.; Krichevsky A. M.; Breakefield X. O. Glioblastoma microvesicles transport RNA and proteins that promote tumour growth and provide diagnostic biomarkers. Nat. Cell Biol. 2008, 10 (12), 1470–1476. 10.1038/ncb1800. 19011622 PMC3423894

[ref6] Graner M. W.; Alzate O.; Dechkovskaia A. M.; Keene J. D.; Sampson J. H.; Mitchell D. A.; Bigner D. D. Proteomic and immunologic analyses of brain tumor exosomes. FASEB J. 2009, 23 (5), 1541–57. 10.1096/fj.08-122184. 19109410 PMC2669426

[ref7] Simpson R. J.; Lim J. W.; Moritz R. L.; Mathivanan S. Exosomes: proteomic insights and diagnostic potential. Expert Rev. Proteomics 2009, 6 (3), 267–83. 10.1586/epr.09.17. 19489699

[ref8] Mathivanan S.; Fahner C. J.; Reid G. E.; Simpson R. J. ExoCarta 2012: database of exosomal proteins, RNA and lipids. Nucleic Acids Res. 2012, 40 (D1), D1241–D1244. 10.1093/nar/gkr828. 21989406 PMC3245025

[ref9] Whiteside T. L. Tumor-Derived Exosomes and Their Role in Cancer Progression. Adv. Clin Chem. 2016, 74, 103–41. 10.1016/bs.acc.2015.12.005. 27117662 PMC5382933

[ref10] Suchorska W. M.; Lach M. S. The role of exosomes in tumor progression and metastasis (Review). Oncol. Rep. 2016, 35 (3), 1237–44. 10.3892/or.2015.4507. 26707854

[ref11] Liu Y.; Cao X. Organotropic metastasis: role of tumor exosomes. Cell Res. 2016, 26 (2), 149–50. 10.1038/cr.2015.153. 26704450 PMC4746605

[ref12] Xiao Y.; Zhong J.; Zhong B.; Huang J.; Jiang L.; Jiang Y.; Yuan J.; Sun J.; Dai L.; Yang C.; Li Z.; Wang J.; Zhong T. Exosomes as potential sources of biomarkers in colorectal cancer. Cancer Lett. 2020, 476, 13–22. 10.1016/j.canlet.2020.01.033. 32044357

[ref13] Ha D.; Yang N.; Nadithe V. Exosomes as therapeutic drug carriers and delivery vehicles across biological membranes: current perspectives and future challenges. Acta Pharm. Sin B 2016, 6 (4), 287–96. 10.1016/j.apsb.2016.02.001. 27471669 PMC4951582

[ref14] Lai R. C.; Yeo R. W.; Tan K. H.; Lim S. K. Exosomes for drug delivery - a novel application for the mesenchymal stem cell. Biotechnol Adv. 2013, 31 (5), 543–51. 10.1016/j.biotechadv.2012.08.008. 22959595

[ref15] Aqil F.; Munagala R.; Jeyabalan J.; Agrawal A. K.; Kyakulaga A. H.; Wilcher S. A.; Gupta R. C. Milk exosomes - Natural nanoparticles for siRNA delivery. Cancer Lett. 2019, 449, 186–195. 10.1016/j.canlet.2019.02.011. 30771430

[ref16] Minciacchi V. R.; Freeman M. R.; Di Vizio D. Extracellular vesicles in cancer: exosomes, microvesicles and the emerging role of large oncosomes. Semin Cell Dev Biol. 2015, 40, 41–51. 10.1016/j.semcdb.2015.02.010. 25721812 PMC4747631

[ref17] Record M. Intercellular communication by exosomes in placenta: a possible role in cell fusion?. Placenta 2014, 35 (5), 297–302. 10.1016/j.placenta.2014.02.009. 24661568

[ref18] Yellon D. M.; Davidson S. M. Exosomes: nanoparticles involved in cardioprotection?. Circ. Res. 2014, 114 (2), 325–32. 10.1161/CIRCRESAHA.113.300636. 24436428

[ref19] Henne W. M.; Buchkovich N. J.; Emr S. D. The ESCRT pathway. Dev Cell 2011, 21 (1), 77–91. 10.1016/j.devcel.2011.05.015. 21763610

[ref20] Hurley J. H. ESCRTs are everywhere. EMBO J. 2015, 34 (19), 2398–407. 10.15252/embj.201592484. 26311197 PMC4601661

[ref21] Villarroya-Beltri C.; Baixauli F.; Gutierrez-Vazquez C.; Sanchez-Madrid F.; Mittelbrunn M. Sorting it out: regulation of exosome loading. Semin Cancer Biol. 2014, 28, 3–13. 10.1016/j.semcancer.2014.04.009. 24769058 PMC4640178

[ref22] Airola M. V.; Hannun Y. A. Sphingolipid metabolism and neutral sphingomyelinases. Handb Exp Pharmacol 2013, 215 (215), 57–76. 10.1007/978-3-7091-1368-4_3. PMC4043343 23579449

[ref23] Castro B. M.; Prieto M.; Silva L. C. Ceramide: a simple sphingolipid with unique biophysical properties. Prog. Lipid Res. 2014, 54, 53–67. 10.1016/j.plipres.2014.01.004. 24513486

[ref24] Zhang Y.; Liu Y.; Liu H.; Tang W. H. Exosomes: biogenesis, biologic function and clinical potential. Cell Biosci. 2019, 9, 19 10.1186/s13578-019-0282-2. 30815248 PMC6377728

[ref25] Batrakova E. V.; Kim M. S. Using exosomes, naturally-equipped nanocarriers, for drug delivery. J. Controlled Release 2015, 219, 396–405. 10.1016/j.jconrel.2015.07.030. PMC4656109 26241750

[ref26] Hoshino A.; Costa-Silva B.; Shen T. L.; Rodrigues G.; Hashimoto A.; Tesic Mark M.; Molina H.; Kohsaka S.; Di Giannatale A.; Ceder S.; Singh S.; Williams C.; Soplop N.; Uryu K.; Pharmer L.; King T.; Bojmar L.; Davies A. E.; Ararso Y.; Zhang T.; Zhang H.; Hernandez J.; Weiss J. M.; Dumont-Cole V. D.; Kramer K.; Wexler L. H.; Narendran A.; Schwartz G. K.; Healey J. H.; Sandstrom P.; Labori K. J.; Kure E. H.; Grandgenett P. M.; Hollingsworth M. A.; de Sousa M.; Kaur S.; Jain M.; Mallya K.; Batra S. K.; Jarnagin W. R.; Brady M. S.; Fodstad O.; Muller V.; Pantel K.; Minn A. J.; Bissell M. J.; Garcia B. A.; Kang Y.; Rajasekhar V. K.; Ghajar C. M.; Matei I.; Peinado H.; Bromberg J.; Lyden D. Tumour exosome integrins determine organotropic metastasis. Nature 2015, 527 (7578), 329–335. 10.1038/nature15756. 26524530 PMC4788391

[ref27] Laulagnier K.; Vincent-Schneider H.; Hamdi S.; Subra C.; Lankar D.; Record M. Characterization of exosome subpopulations from RBL-2H3 cells using fluorescent lipids. Blood Cells Mol. Dis 2005, 35 (2), 116–21. 10.1016/j.bcmd.2005.05.010. 16023874

[ref28] Subra C.; Laulagnier K.; Perret B.; Record M. Exosome lipidomics unravels lipid sorting at the level of multivesicular bodies. Biochimie 2007, 89 (2), 205–12. 10.1016/j.biochi.2006.10.014. 17157973

[ref29] Dou Y.; Cha D. J.; Franklin J. L.; Higginbotham J. N.; Jeppesen D. K.; Weaver A. M.; Prasad N.; Levy S.; Coffey R. J.; Patton J. G.; Zhang B. Circular RNAs are down-regulated in KRAS mutant colon cancer cells and can be transferred to exosomes. Sci. Rep 2016, 6, 37982 10.1038/srep37982. 27892494 PMC5125100

[ref30] Rabinowits G.; Gercel-Taylor C.; Day J. M.; Taylor D. D.; Kloecker G. H. Exosomal microRNA: a diagnostic marker for lung cancer. Clin Lung Cancer 2009, 10 (1), 42–6. 10.3816/CLC.2009.n.006. 19289371

[ref31] Yuan T.; Huang X.; Woodcock M.; Du M.; Dittmar R.; Wang Y.; Tsai S.; Kohli M.; Boardman L.; Patel T.; Wang L. Plasma extracellular RNA profiles in healthy and cancer patients. Sci. Rep 2016, 6, 19413 10.1038/srep19413. 26786760 PMC4726401

[ref32] Abels E. R.; Breakefield X. O. Introduction to Extracellular Vesicles: Biogenesis, RNA Cargo Selection, Content, Release, and Uptake. Cell Mol. Neurobiol 2016, 36 (3), 301–12. 10.1007/s10571-016-0366-z. 27053351 PMC5546313

[ref33] Gezer U.; Özgür E.; Cetinkaya M.; Isin M.; Dalay N. Long non-coding RNAs with low expression levels in cells are enriched in secreted exosomes. Cell Biol. Int. 2014, 38 (9), 1076–1079. 10.1002/cbin.10301. 24798520

[ref34] Jiang Y.; Wang F.; Wang K.; Zhong Y.; Wei X.; Wang Q.; Zhang H. Engineered Exosomes: A Promising Drug Delivery Strategy for Brain Diseases. Curr. Med. Chem. 2022, 29 (17), 3111–3124. 10.2174/0929867328666210902142015. 34477508

[ref35] Goh C. Y.; Wyse C.; Ho M.; O’Beirne E.; Howard J.; Lindsay S.; Kelly P.; Higgins M.; McCann A. Exosomes in triple negative breast cancer: Garbage disposals or Trojan horses?. Cancer Lett. 2020, 473, 90–97. 10.1016/j.canlet.2019.12.046. 31904485

[ref36] Zhou X.; Li T.; Chen Y.; Zhang N.; Wang P.; Liang Y.; Long M.; Liu H.; Mao J.; Liu Q.; Sun X.; Chen H. Mesenchymal stem cellderived extracellular vesicles promote the in vitro proliferation and migration of breast cancer cells through the activation of the ERK pathway. Int. J. Oncol. 2019, 54 (5), 1843–1852. 10.3892/ijo.2019.4747. 30864702

[ref37] Wang S.; Li F.; Ye T.; Wang J.; Lyu C.; Qing S.; Ding Z.; Gao X.; Jia R.; Yu D.; Ren J.; Wei W.; Ma G. Macrophage-tumor chimeric exosomes accumulate in lymph node and tumor to activate the immune response and the tumor microenvironment. Sci. Transl Med. 2021, 13 (615), eabb6981 10.1126/scitranslmed.abb6981. 34644149

[ref38] Lin S.; Yu Z.; Chen D.; Wang Z.; Miao J.; Li Q.; Zhang D.; Song J.; Cui D. Progress in Microfluidics-Based Exosome Separation and Detection Technologies for Diagnostic Applications. Small 2020, 16 (9), 1903916 10.1002/smll.201903916. 31663295

[ref39] Chen L.; Wang L.; Zhu L.; Xu Z.; Liu Y.; Li Z.; Zhou J.; Luo F. Exosomes as Drug Carriers in Anti-Cancer Therapy. Front Cell Dev Biol. 2022, 10, 728616 10.3389/fcell.2022.728616. 35155421 PMC8826094

[ref40] Ding L.; Yang X.; Gao Z.; Effah C. Y.; Zhang X.; Wu Y.; Qu L. A Holistic Review of the State-of-the-Art Microfluidics for Exosome Separation: An Overview of the Current Status, Existing Obstacles, and Future Outlook. Small 2021, 17 (29), 2007174 10.1002/smll.202007174. 34047052

[ref41] Mohammadi M.; Zargartalebi H.; Salahandish R.; Aburashed R.; Wey Yong K.; Sanati-Nezhad A. Emerging technologies and commercial products in exosome-based cancer diagnosis and prognosis. Biosens Bioelectron 2021, 183, 113176 10.1016/j.bios.2021.113176. 33845291

[ref42] Narayanan E. Exosomes as drug delivery vehicles for cancer treatment. Current Nanoscience 2020, 16 (1), 15–26. 10.2174/1573413715666190219112422.

[ref43] Wang J.; Ma P.; Kim D. H.; Liu B. F.; Demirci U. Towards Microfluidic-Based Exosome Isolation and Detection for Tumor Therapy. Nano Today 2021, 37, 101066 10.1016/j.nantod.2020.101066. 33777166 PMC7990116

[ref44] Wan Y.; Cheng G.; Liu X.; Hao S.-J.; Nisic M.; Zhu C.-D.; Xia Y.-Q.; Li W.-Q.; Wang Z.-G.; Zhang W.-L.; Rice S. J.; Sebastian A.; Albert I.; Belani C. P.; Zheng S.-Y. Rapid magnetic isolation of extracellular vesicles via lipid-based nanoprobes. Nat. Biomed Eng. 2017, 1, 0058 10.1038/s41551-017-0058. 28966872 PMC5618714

[ref45] Wan Y.; Maurer M.; He H. Z.; et al. Enrichment of extracellular vesicles with lipid nanoprobe functionalized nanostructured silica. Lab Chip 2019, 19 (14), 2346–2355. 10.1039/C8LC01359D. 31232418 PMC6669184

[ref46] Dolatmoradi A.; Mirtaheri E.; El-Zahab B. Thermo-acoustofluidic separation of vesicles based on cholesterol content. Lab Chip 2017, 17 (7), 1332–1339. 10.1039/C7LC00161D. 28272605

[ref47] Wu M.; Ozcelik A.; Rufo J.; Wang Z.; Fang R.; Jun Huang T. Acoustofluidic separation of cells and particles. Microsyst . Nanoeng. 2019, 5, 32 10.1038/s41378-019-0064-3. 31231539 PMC6545324

[ref48] Zhang J.; Ji C.; Zhang H.; Shi H.; Mao F.; Qian H.; Xu W.; Wang D.; Pan J.; Fang X.; Santos H. A.; Zhang X. Engineered neutrophil-derived exosome-like vesicles for targeted cancer therapy. Sci. Adv. 2022, 8 (2), eabj8207 10.1126/sciadv.abj8207. 35020437 PMC8754405

[ref49] Wee I.; Syn N.; Sethi G.; Goh B. C.; Wang L. Role of tumor-derived exosomes in cancer metastasis. Biochim Biophys Acta Rev. Cancer 2019, 1871 (1), 12–19. 10.1016/j.bbcan.2018.10.004. 30419312

[ref50] Jafari A.; Babajani A.; Abdollahpour-Alitappeh M.; Ahmadi N.; Rezaei-Tavirani M. Exosomes and cancer: from molecular mechanisms to clinical applications. Med. Oncol. 2021, 38, 45 10.1007/s12032-021-01491-0. 33743101

[ref51] Huang Y. K.; Wang M.; Sun Y.; Di Costanzo N.; Mitchell C.; Achuthan A.; Hamilton J. A.; Busuttil R. A.; Boussioutas A. Macrophage spatial heterogeneity in gastric cancer defined by multiplex immunohistochemistry. Nat. Commun. 2019, 10, 3928 10.1038/s41467-019-11788-4. 31477692 PMC6718690

[ref52] Lin X.; Wang S.; Sun M.; Zhang C.; Wei C.; Yang C.; Dou R.; Liu Q.; Xiong B. Correction to: miR-195–5p/NOTCH2-mediated EMT modulates IL-4 secretion in colorectal cancer to affect M2-like TAM polarization. J. Hematol. Oncol. 2019, 12, 122 10.1186/s13045-019-0810-x. 31757211 PMC6874810

[ref53] Han C.; Zhang C.; Wang H.; Zhao L. Exosome-mediated communication between tumor cells and tumor-associated macrophages: implications for tumor microenvironment. Oncoimmunology 2021, 10 (1), 1887552 10.1080/2162402X.2021.1887552. 33680573 PMC7901554

[ref54] Kumar A.; Deep G. Hypoxia in tumor microenvironment regulates exosome biogenesis: Molecular mechanisms and translational opportunities. Cancer Lett. 2020, 479, 23–30. 10.1016/j.canlet.2020.03.017. 32201202

[ref55] Park J. E.; Dutta B.; Tse S. W.; Gupta N.; Tan C. F.; Low J. K.; Yeoh K. W.; Kon O. L.; Tam J. P.; Sze S. K. Hypoxia-induced tumor exosomes promote M2-like macrophage polarization of infiltrating myeloid cells and microRNA-mediated metabolic shift. Oncogene 2019, 38 (26), 5158–5173. 10.1038/s41388-019-0782-x. 30872795

[ref56] Luan Y.; Li X.; Luan Y.; Zhao R.; Li Y.; Liu L.; Hao Y.; Oleg Vladimir B.; Jia L. Circulating lncRNA UCA1 Promotes Malignancy of Colorectal Cancer via the miR-143/MYO6 Axis. Mol. Ther Nucleic Acids 2020, 19, 790–803. 10.1016/j.omtn.2019.12.009. 31955010 PMC6970172

[ref57] Li Z.; Qin X.; Bian W.; Li Y.; Shan B.; Yao Z.; Li S. Exosomal lncRNA ZFAS1 regulates esophageal squamous cell carcinoma cell proliferation, invasion, migration and apoptosis via microRNA-124/STAT3 axis. J. Exp. Clin. Cancer Res. 2019, 38, 477 10.1186/s13046-019-1473-8. 31775815 PMC6882153

[ref58] Soltesz B.; Buglyo G.; Nemeth N.; Szilagyi M.; Pos O.; Szemes T.; Balogh I.; Nagy B. The Role of Exosomes in Cancer Progression. Int. J. Mol. Sci. 2022, 23 (1), 8 10.3390/ijms23010008. PMC8744561 35008434

[ref59] Munshi A.; Mehic J.; Creskey M.; Gobin J.; Gao J.; Rigg E.; Muradia G.; Luebbert C. C.; Westwood C.; Stalker A.; Allan D. S.; Johnston M. J. W.; Cyr T.; Rosu-Myles M.; Lavoie J. R. A comprehensive proteomics profiling identifies NRP1 as a novel identity marker of human bone marrow mesenchymal stromal cell-derived small extracellular vesicles. Stem Cell Res. Ther. 2019, 10, 401 10.1186/s13287-019-1516-2. 31852509 PMC6921509

[ref60] St-Denis-Bissonnette F.; Khoury R.; Mediratta K.; El-Sahli S.; Wang L.; Lavoie J. R. Applications of Extracellular Vesicles in Triple-Negative Breast Cancer. Cancers (Basel) 2022, 14 (2), 451 10.3390/cancers14020451. 35053616 PMC8773485

[ref61] Ramirez-Ricardo J.; Leal-Orta E.; Martinez-Baeza E.; Ortiz-Mendoza C.; Breton-Mora F.; Herrera-Torres A.; Elizalde-Acosta I.; Cortes-Reynosa P.; Thompson-Bonilla R.; Perez Salazar E. Circulating extracellular vesicles from patients with breast cancer enhance migration and invasion via a Srcdependent pathway in MDAMB231 breast cancer cells. Mol. Med. Rep 2020, 22 (3), 1932–1948. 10.3892/mmr.2020.11259. 32582965 PMC7411406

[ref62] Rabe D. C.; Walker N. D.; Rustandy F. D.; Wallace J.; Lee J.; Stott S. L.; Rosner M. R. Tumor Extracellular Vesicles Regulate Macrophage-Driven Metastasis through CCL5. Cancers 2021, 13 (14), 3459 10.3390/cancers13143459. 34298673 PMC8303898

[ref63] Wang H. X.; Gires O. Tumor-derived extracellular vesicles in breast cancer: From bench to bedside. Cancer Lett. 2019, 460, 54–64. 10.1016/j.canlet.2019.06.012. 31233837

[ref64] Yoshikawa M.; Iinuma H.; Umemoto Y.; Yanagisawa T.; Matsumoto A.; Jinno H. Exosome-encapsulated microRNA-223–3p as a minimally invasive biomarker for the early detection of invasive breast cancer. Oncol Lett. 2018, 15 (6), 9584–9592. 10.3892/ol.2018.8457. 29805680 PMC5958689

[ref65] Sun S.; Chen H.; Xu C.; et al. Exosomal miR-106b serves as a novel marker for lung cancer and promotes cancer metastasis via targeting PTEN. Life Sci. 2020, 244, 117297 10.1016/j.lfs.2020.117297. 31954745

[ref66] Tian Y.; Ma L.; Gong M.; et al. Protein Profiling and Sizing of Extracellular Vesicles from Colorectal Cancer Patients via Flow Cytometry. ACS Nano 2018, 12 (1), 671–680. 10.1021/acsnano.7b07782. 29300458

[ref67] Rodriguez M.; Bajo-Santos C.; Hessvik N. P.; Lorenz S.; Fromm B.; Berge V.; Sandvig K.; Line̅ A.; Llorente A.; et al. Identification of non-invasive miRNAs biomarkers for prostate cancer by deep sequencing analysis of urinary exosomes. Mol. Cancer 2017, 16, 156 10.1186/s12943-017-0726-4. 28982366 PMC5629793

[ref68] Huang X.-Y.; Huang Z.-L.; Huang J.; Xu B.; Huang X.-Y.; Xu Y.-H.; Zhou J.; Tang Z.-Y.; et al. Exosomal circRNA-100338 promotes hepatocellular carcinoma metastasis via enhancing invasiveness and angiogenesis. J. Exp. Clin. Cancer Res. 2020, 39, 20 10.1186/s13046-020-1529-9. 31973767 PMC6979009

[ref69] Elkhoury K.; Kocak P.; Kang A.; Arab-Tehrany E.; Ellis Ward J.; Shin S. R. Engineering Smart Targeting Nanovesicles and Their Combination with Hydrogels for Controlled Drug Delivery. Pharmaceutics 2020, 12 (9), 849 10.3390/pharmaceutics12090849. 32906833 PMC7559099

[ref70] Butreddy A.; Kommineni N.; Dudhipala N. Exosomes as Naturally Occurring Vehicles for Delivery of Biopharmaceuticals: Insights from Drug Delivery to Clinical Perspectives. Nanomaterials 2021, 11 (6), 1481 10.3390/nano11061481. 34204903 PMC8229362

[ref71] Vázquez-Ríos A. J.; Molina-Crespo Á.; Bouzo B. L.; López-López R.; Moreno-Bueno G.; de la Fuente M. Exosome-mimetic nanoplatforms for targeted cancer drug delivery. J. Nanobiotechnology 2019, 17, 85 10.1186/s12951-019-0517-8. 31319859 PMC6637649

[ref72] Kibria G.; Ramos E. K.; Wan Y.; Gius D. R.; Liu H. Exosomes as a Drug Delivery System in Cancer Therapy: Potential and Challenges. Mol. Pharmaceutics 2018, 15 (9), 3625–3633. 10.1021/acs.molpharmaceut.8b00277. PMC6546090 29771531

[ref73] Shao J.; Zaro J.; Shen Y. Advances in Exosome-Based Drug Delivery and Tumor Targeting: From Tissue Distribution to Intracellular Fate. Int. J. Nanomedicine 2020, 15, 9355–9371. 10.2147/IJN.S281890. 33262592 PMC7700079

[ref74] Gutierrez-Millan C.; Calvo Díaz C.; Lanao J. M.; Colino C. I. Advances in exosomes-based drug delivery systems. Macromol. Biosci. 2021, 21 (1), 2000269 10.1002/mabi.202000269. 33094544

[ref75] Kučuk N.; Primožič M.; Knez Ž.; Leitgeb M. Exosomes Engineering and Their Roles as Therapy Delivery Tools, Therapeutic Targets, and Biomarkers. Int. J. Mol. Sci. 2021, 22 (17), 9543 10.3390/ijms22179543. 34502452 PMC8431173

[ref76] Vader P.; Mol E. A.; Pasterkamp G.; Schiffelers R. M. Extracellular vesicles for drug delivery. Adv. Drug Deliv. Rev. 2016, 106, 148–156. 10.1016/j.addr.2016.02.006. 26928656

[ref77] Akuma P.; Okagu O. D.; Udenigwe C. C. Naturally occurring exosome vesicles as potential delivery vehicle for bioactive compounds. Front. Sustain. Food Syst. 2019, 3, 23 10.3389/fsufs.2019.00023.

[ref78] Butreddy A.; Kommineni N.; Dudhipala N. Exosomes as Naturally Occurring Vehicles for Delivery of Biopharmaceuticals: Insights from Drug Delivery to Clinical Perspectives. Nanomaterials 2021, 11 (6), 1481 10.3390/nano11061481. 34204903 PMC8229362

[ref79] Fuhrmann G.; Serio A.; Mazo M.; Nair R.; Stevens M. M. Active loading into extracellular vesicles significantly improves the cellular uptake and photodynamic effect of porphyrins. J. Controlled Release 2015, 205, 35–44. 10.1016/j.jconrel.2014.11.029. 25483424

[ref80] Kim M. S.; Haney M. J.; Zhao Y.; Mahajan V.; Deygen I.; Klyachko N. L.; Inskoe E.; Piroyan A.; Sokolsky M.; Okolie O.; Hingtgen S. D.; Kabanov A. V.; Batrakova E. V. Development of exosome-encapsulated paclitaxel to overcome MDR in cancer cells. Nanomedicine 2016, 12 (3), 655–664. 10.1016/j.nano.2015.10.012. 26586551 PMC4809755

[ref81] Sato Y. T.; Umezaki K.; Sawada S.; Mukai S. A.; Sasaki Y.; Harada N.; Shiku H.; Akiyoshi K. Engineering hybrid exosomes by membrane fusion with liposomes. Sci. Rep 2016, 6, 21933 10.1038/srep21933. 26911358 PMC4766490

[ref82] Haney M. J.; Klyachko N. L.; Zhao Y.; Gupta R.; Plotnikova E. G.; He Z.; Patel T.; Piroyan A.; Sokolsky M.; Kabanov A. V.; Batrakova E. V. Exosomes as drug delivery vehicles for Parkinson’s disease therapy. J. Controlled Release 2015, 207, 18–30. 10.1016/j.jconrel.2015.03.033. PMC4430381 25836593

[ref83] Luan Y.; Li X.; Luan Y.; Zhao R.; Li Y.; Liu L.; Hao Y.; Oleg Vladimir B.; Jia L. Circulating lncRNA UCA1 Promotes Malignancy of Colorectal Cancer via the miR-143/MYO6 Axis. Mol. Ther Nucleic Acids 2020, 19, 790–803. 10.1016/j.omtn.2019.12.009. 31955010 PMC6970172

[ref84] Kim H.; Jang H.; Cho H.; Choi J.; Hwang K. Y.; Choi Y.; Kim S. H.; Yang Y. Recent Advances in Exosome-Based Drug Delivery for Cancer Therapy. Cancers 2021, 13 (17), 4435 10.3390/cancers13174435. 34503245 PMC8430743

[ref85] Gurung S.; Perocheau D.; Touramanidou L.; Baruteau J. The exosome journey: from biogenesis to uptake and intracellular signalling. Cell Commun. Signal 2021, 19, 47 10.1186/s12964-021-00730-1. 33892745 PMC8063428

[ref86] Camussi G.; Deregibus M. C.; Bruno S.; Grange C.; Fonsato V.; Tetta C. Exosome/microvesicle-mediated epigenetic reprogramming of cells. Am. J. Cancer Res. 2011, 1 (1), 98–110. 21969178 PMC3180104

[ref87] Bruno S.; Grange C.; Deregibus M. C.; Calogero R. A.; Saviozzi S.; Collino F.; Morando L.; Busca A.; Falda M.; Bussolati B.; Tetta C.; Camussi G. Mesenchymal stem cell-derived microvesicles protect against acute tubular injury. J. Am. Soc. Nephrol 2009, 20 (5), 1053–67. 10.1681/ASN.2008070798. 19389847 PMC2676194

[ref88] Elsharkasy O. M.; Nordin J. Z.; Hagey D. W.; de Jong O. G.; Schiffelers R. M.; Andaloussi S. E.; Vader P. Extracellular vesicles as drug delivery systems: Why and how?. Adv. Drug Deliv Rev. 2020, 159, 332–343. 10.1016/j.addr.2020.04.004. 32305351

[ref89] Armstrong J. P.; Holme M. N.; Stevens M. M. Re-Engineering Extracellular Vesicles as Smart Nanoscale Therapeutics. ACS Nano 2017, 11 (1), 69–83. 10.1021/acsnano.6b07607. 28068069 PMC5604727

[ref90] Lara P.; Chan A. B.; Cruz L. J.; Quest A. F. G.; Kogan M. J. Exploiting the Natural Properties of Extracellular Vesicles in Targeted Delivery towards Specific Cells and Tissues. Pharmaceutics 2020, 12 (11), 1022 10.3390/pharmaceutics12111022. 33114492 PMC7692617

[ref91] Sun D.; Zhuang X.; Xiang X.; Liu Y.; Zhang S.; Liu C.; Barnes S.; Grizzle W.; Miller D.; Zhang H. G. A novel nanoparticle drug delivery system: the anti-inflammatory activity of curcumin is enhanced when encapsulated in exosomes. Mol. Ther 2010, 18 (9), 1606–14. 10.1038/mt.2010.105. 20571541 PMC2956928

[ref92] Banks W. A.; Sharma P.; Bullock K. M.; Hansen K. M.; Ludwig N.; Whiteside T. L. Transport of Extracellular Vesicles across the Blood-Brain Barrier: Brain Pharmacokinetics and Effects of Inflammation. Int. J. Mol. Sci. 2020, 21 (12), 4407 10.3390/ijms21124407. 32575812 PMC7352415

[ref93] Wu X.; Zheng T.; Zhang B. Exosomes in Parkinson’s Disease. Neurosci Bull. 2017, 33 (3), 331–338. 10.1007/s12264-016-0092-z. 28025780 PMC5567508

[ref94] Choi H.; Yim H.; Park C.; Ahn S. H.; Ahn Y.; Lee A.; Yang H.; Choi C. Targeted Delivery of Exosomes Armed with Anti-Cancer Therapeutics. Membranes 2022, 12 (1), 85 10.3390/membranes12010085. 35054611 PMC8782002

[ref95] Zhang J.; Li S.; Li L.; Li M.; Guo C.; Yao J.; Mi S. Exosome and exosomal microRNA: trafficking, sorting, and function. Genomics Proteomics Bioinformatics 2015, 13 (1), 17–24. 10.1016/j.gpb.2015.02.001. 25724326 PMC4411500

[ref96] Sun Z.; Shi K.; Yang S.; Liu J.; Zhou Q.; Wang G.; Song J.; Li Z.; Zhang Z.; Yuan W. Effect of exosomal miRNA on cancer biology and clinical applications. Mol. Cancer 2018, 17, 147 10.1186/s12943-018-0897-7. 30309355 PMC6182840

[ref97] Kowal J.; Tkach M.; Thery C. Biogenesis and secretion of exosomes. Curr. Opin Cell Biol. 2014, 29, 116–25. 10.1016/j.ceb.2014.05.004. 24959705

[ref98] Tai Y. L.; Chen K. C.; Hsieh J. T.; Shen T. L. Exosomes in cancer development and clinical applications. Cancer Sci. 2018, 109 (8), 2364–2374. 10.1111/cas.13697. 29908100 PMC6113508

[ref99] Becker A.; Thakur B. K.; Weiss J. M.; Kim H. S.; Peinado H.; Lyden D. Extracellular Vesicles in Cancer: Cell-to-Cell Mediators of Metastasis. Cancer Cell 2016, 30 (6), 836–848. 10.1016/j.ccell.2016.10.009. 27960084 PMC5157696

[ref100] Quah B. J.; O’Neill H. C. The immunogenicity of dendritic cell-derived exosomes. Blood Cells Mol. Dis 2005, 35 (2), 94–110. 10.1016/j.bcmd.2005.05.002. 15975838

[ref101] Zheng M.; Huang M.; Ma X.; Chen H.; Gao X. Harnessing Exosomes for the Development of Brain Drug Delivery Systems. Bioconjug Chem. 2019, 30 (4), 994–1005. 10.1021/acs.bioconjchem.9b00085. 30855944

[ref102] Jiang B.; Kauffman A. E.; Li L.; McFee W.; Cai B.; Weinstein J.; Lead J. R.; Chatterjee S.; Scott G. I.; Xiao S. Health impacts of environmental contamination of micro- and nanoplastics: a review. Environ. Health Prev. Med. 2020, 25, 29 10.1186/s12199-020-00870-9. 32664857 PMC7362455

[ref103] Tian Y.; Li S.; Song J.; Ji T.; Zhu M.; Anderson G. J.; Wei J.; Nie G. A doxorubicin delivery platform using engineered natural membrane vesicle exosomes for targeted tumor therapy. Biomaterials 2014, 35 (7), 2383–90. 10.1016/j.biomaterials.2013.11.083. 24345736

[ref104] Bai J.; Duan J.; Liu R.; Du Y.; Luo Q.; Cui Y.; Su Z.; Xu J.; Xie Y.; Lu W. Engineered targeting tLyp-1 exosomes as gene therapy vectors for efficient delivery of siRNA into lung cancer cells. Asian J. Pharm. Sci. 2020, 15 (4), 461–471. 10.1016/j.ajps.2019.04.002. 32952669 PMC7486479

[ref105] Bellavia D.; Raimondo S.; Calabrese G.; Forte S.; Cristaldi M.; Patinella A.; Memeo L.; Manno M.; Raccosta S.; Diana P.; Cirrincione G.; Giavaresi G.; Monteleone F.; Fontana S.; De Leo G.; Alessandro R. Interleukin 3- receptor targeted exosomes inhibit in vitro and in vivo Chronic Myelogenous Leukemia cell growth. Theranostics 2017, 7 (5), 1333–1345. 10.7150/thno.17092. 28435469 PMC5399597

[ref106] Liang G.; Zhu Y.; Ali D. J.; Tian T.; Xu H.; Si K.; Sun B.; Chen B.; Xiao Z. Engineered exosomes for targeted co-delivery of miR-21 inhibitor and chemotherapeutics to reverse drug resistance in colon cancer. J. Nanobiotechnol. 2020, 18, 10 10.1186/s12951-019-0563-2. PMC6950820 31918721

[ref107] Limoni S. K.; Moghadam M. F.; Moazzeni S. M.; Gomari H.; Salimi F. Engineered Exosomes for Targeted Transfer of siRNA to HER2 Positive Breast Cancer Cells. Appl. Biochem. Biotechnol. 2019, 187 (1), 352–364. 10.1007/s12010-018-2813-4. 29951961

[ref108] Ohno S.; Takanashi M.; Sudo K.; Ueda S.; Ishikawa A.; Matsuyama N.; Fujita K.; Mizutani T.; Ohgi T.; Ochiya T.; Gotoh N.; Kuroda M. Systemically injected exosomes targeted to EGFR deliver antitumor microRNA to breast cancer cells. Mol. Ther 2013, 21 (1), 185–91. 10.1038/mt.2012.180. 23032975 PMC3538304

[ref109] Cheng Q.; Shi X.; Han M.; Smbatyan G.; Lenz H. J.; Zhang Y. Reprogramming Exosomes as Nanoscale Controllers of Cellular Immunity. J. Am. Chem. Soc. 2018, 140 (48), 16413–16417. 10.1021/jacs.8b10047. 30452238 PMC6469991

[ref110] Liang G.; Kan S.; Zhu Y.; Feng S.; Feng W.; Gao S. Engineered exosome-mediated delivery of functionally active miR-26a and its enhanced suppression effect in HepG2 cells. Int. J. Nanomedicine 2018, 13, 585–599. 10.2147/IJN.S154458. 29430178 PMC5796471

[ref111] Kanuma T.; Yamamoto T.; Kobiyama K.; Moriishi E.; Masuta Y.; Kusakabe T.; Ozasa K.; Kuroda E.; Jounai N.; Ishii K. J. CD63-Mediated Antigen Delivery into Extracellular Vesicles via DNA Vaccination Results in Robust CD8(+) T Cell Responses. J. Immunol 2017, 198 (12), 4707–4715. 10.4049/jimmunol.1600731. 28507029

[ref112] Rountree R. B.; Mandl S. J.; Nachtwey J. M.; Dalpozzo K.; Do L.; Lombardo J. R.; Schoonmaker P. L.; Brinkmann K.; Dirmeier U.; Laus R.; Delcayre A. Exosome targeting of tumor antigens expressed by cancer vaccines can improve antigen immunogenicity and therapeutic efficacy. Cancer Res. 2011, 71 (15), 5235–44. 10.1158/0008-5472.CAN-10-4076. 21670078

[ref113] Wang M.; Altinoglu S.; Takeda Y. S.; Xu Q. Integrating Protein Engineering and Bioorthogonal Click Conjugation for Extracellular Vesicle Modulation and Intracellular Delivery. PLoS One 2015, 10 (11), e0141860 10.1371/journal.pone.0141860. 26529317 PMC4631329

[ref114] Jia G.; Han Y.; An Y.; Ding Y.; He C.; Wang X.; Tang Q. NRP-1 targeted and cargo-loaded exosomes facilitate simultaneous imaging and therapy of glioma in vitro and in vivo. Biomaterials 2018, 178, 302–316. 10.1016/j.biomaterials.2018.06.029. 29982104

[ref115] Kim M. S.; Haney M. J.; Zhao Y.; Yuan D.; Deygen I.; Klyachko N. L.; Kabanov A. V.; Batrakova E. V. Engineering macrophage-derived exosomes for targeted paclitaxel delivery to pulmonary metastases: in vitro and in vivo evaluations. Nanomedicine 2018, 14 (1), 195–204. 10.1016/j.nano.2017.09.011. 28982587

[ref116] Cao Y.; Wu T.; Zhang K.; Meng X.; Dai W.; Wang D.; Dong H.; Zhang X. Engineered Exosome-Mediated Near-Infrared-II Region V_2_C Quantum Dot Delivery for Nucleus-Target Low-Temperature Photothermal Therapy. ACS Nano 2019, 13 (2), 1499–1510. 10.1021/acsnano.8b07224. 30677286

[ref117] Pi F.; Binzel D. W.; Lee T. J.; Li Z.; Sun M.; Rychahou P.; Li H.; Haque F.; Wang S.; Croce C. M.; Guo B.; Evers B. M.; Guo P. Nanoparticle orientation to control RNA loading and ligand display on extracellular vesicles for cancer regression. Nat. Nanotechnol 2018, 13 (1), 82–89. 10.1038/s41565-017-0012-z. 29230043 PMC5762263

[ref118] Zou J.; Shi M.; Liu X.; Jin C.; Xing X.; Qiu L.; Tan W. Aptamer-Functionalized Exosomes: Elucidating the Cellular Uptake Mechanism and the Potential for Cancer-Targeted Chemotherapy. Anal. Chem. 2019, 91 (3), 2425–2430. 10.1021/acs.analchem.8b05204. 30620179 PMC6662586

[ref119] Wan Y.; Wang L.; Zhu C.; Zheng Q.; Wang G.; Tong J.; Fang Y.; Xia Y.; Cheng G.; He X.; Zheng S. Y. Aptamer-Conjugated Extracellular Nanovesicles for Targeted Drug Delivery. Cancer Res. 2018, 78 (3), 798–808. 10.1158/0008-5472.CAN-17-2880. 29217761 PMC5811376

[ref120] Ye Z.; Zhang T.; He W.; Jin H.; Liu C.; Yang Z.; Ren J. Methotrexate-Loaded Extracellular Vesicles Functionalized with Therapeutic and Targeted Peptides for the Treatment of Glioblastoma Multiforme. ACS Appl. Mater. Interfaces 2018, 10 (15), 12341–12350. 10.1021/acsami.7b18135. 29564886

[ref121] Cheng H.; Fan J. H.; Zhao L. P.; Fan G. L.; Zheng R. R.; Qiu X. Z.; Yu X. Y.; Li S. Y.; Zhang X. Z. Chimeric peptide engineered exosomes for dual-stage light guided plasma membrane and nucleus targeted photodynamic therapy. Biomaterials 2019, 211, 14–24. 10.1016/j.biomaterials.2019.05.004. 31078049

[ref122] Koh E.; Lee E. J.; Nam G. H.; Hong Y.; Cho E.; Yang Y.; Kim I. S. Exosome-SIRPalpha, a CD47 blockade increases cancer cell phagocytosis. Biomaterials 2017, 121, 121–129. 10.1016/j.biomaterials.2017.01.004. 28086180

[ref123] Wang J.; Li W.; Lu Z.; Zhang L.; Hu Y.; Li Q.; Du W.; Feng X.; Jia H.; Liu B. F. The use of RGD-engineered exosomes for enhanced targeting ability and synergistic therapy toward angiogenesis. Nanoscale 2017, 9 (40), 15598–15605. 10.1039/C7NR04425A. 28990632

[ref124] Yang Z.; Shi J.; Xie J.; Wang Y.; Sun J.; Liu T.; Zhao Y.; Zhao X.; Wang X.; Ma Y.; Malkoc V.; Chiang C.; Deng W.; Chen Y.; Fu Y.; Kwak K. J.; Fan Y.; Kang C.; Yin C.; Rhee J.; Bertani P.; Otero J.; Lu W.; Yun K.; Lee A. S.; Jiang W.; Teng L.; Kim B. Y. S.; Lee L. J. Large-scale generation of functional mRNA-encapsulating exosomes via cellular nanoporation. Nat. Biomed Eng. 2020, 4 (1), 69–83. 10.1038/s41551-019-0485-1. 31844155 PMC7080209

[ref125] Shi S.; Li T.; Wen X.; Wu S. Y.; Xiong C.; Zhao J.; Lincha V. R.; Chow D. S.; Liu Y.; Sood A. K.; Li C. Copper-64 Labeled PEGylated Exosomes for In Vivo Positron Emission Tomography and Enhanced Tumor Retention. Bioconjug Chem. 2019, 30 (10), 2675–2683. 10.1021/acs.bioconjchem.9b00587. 31560538 PMC6947533

[ref126] Li S.; Wu Y.; Ding F.; Yang J.; Li J.; Gao X.; Zhang C.; Feng J. Engineering macrophage-derived exosomes for targeted chemotherapy of triple-negative breast cancer. Nanoscale 2020, 12 (19), 10854–10862. 10.1039/D0NR00523A. 32396590

[ref127] Ortega A.; Martinez-Arroyo O.; Forner M. J.; Cortes R. Exosomes as Drug Delivery Systems: Endogenous Nanovehicles for Treatment of Systemic Lupus Erythematosus. Pharmaceutics 2021, 13 (1), 3 10.3390/pharmaceutics13010003. PMC7821934 33374908

[ref128] Jiang L.; Gu Y.; Du Y.; Liu J. Exosomes: Diagnostic Biomarkers and Therapeutic Delivery Vehicles for Cancer. Mol. Pharmaceutics 2019, 16 (8), 3333–3349. 10.1021/acs.molpharmaceut.9b00409. 31241965

[ref129] von Minckwitz G.; Huang C.-S.; Mano M. S.; Loibl S.; Mamounas E. P.; Untch M.; Wolmark N.; Rastogi P.; Schneeweiss A.; Redondo A.; Fischer H. H.; Jacot W.; Conlin A. K.; Arce-Salinas C.; Wapnir I. L.; Jackisch C.; DiGiovanna M. P.; Fasching P. A.; Crown J. P.; Wulfing P.; Shao Z.; Rota Caremoli E.; Wu H.; Lam L. H.; Tesarowski D.; Smitt M.; Douthwaite H.; Singel S. M.; Geyer C. E. Trastuzumab Emtansine for Residual Invasive HER2-Positive Breast Cancer. N. Engl. J. Med. 2019, 380 (7), 617–628. 10.1056/NEJMoa1814017. 30516102

[ref130] Schmid P.; Adams S.; Rugo H. S.; Schneeweiss A.; Barrios C. H.; Iwata H.; Dieras V.; Hegg R.; Im S. A.; Shaw Wright G.; Henschel V.; Molinero L.; Chui S. Y.; Funke R.; Husain A.; Winer E. P.; Loi S.; Emens L. A. Atezolizumab and Nab-Paclitaxel in Advanced Triple-Negative Breast Cancer. N. Engl. J. Med. 2018, 379 (22), 2108–2121. 10.1056/NEJMoa1809615. 30345906

[ref131] Im S. A.; Lu Y. S.; Bardia A.; Harbeck N.; Colleoni M.; Franke F.; Chow L.; Sohn J.; Lee K. S.; Campos-Gomez S.; Villanueva-Vazquez R.; Jung K. H.; Chakravartty A.; Hughes G.; Gounaris I.; Rodriguez-Lorenc K.; Taran T.; Hurvitz S.; Tripathy D. Overall Survival with Ribociclib plus Endocrine Therapy in Breast Cancer. N Engl J. Med. 2019, 381 (4), 307–316. 10.1056/NEJMoa1903765. 31166679

[ref132] Horn L.; Mansfield A. S.; Szczesna A.; Havel L.; Krzakowski M.; Hochmair M. J.; Huemer F.; Losonczy G.; Johnson M. L.; Nishio M.; Reck M.; Mok T.; Lam S.; Shames D. S.; Liu J.; Ding B.; Lopez-Chavez A.; Kabbinavar F.; Lin W.; Sandler A.; Liu S. V. First-Line Atezolizumab plus Chemotherapy in Extensive-Stage Small-Cell Lung Cancer. N. Engl. J. Med. 2018, 379 (23), 2220–2229. 10.1056/NEJMoa1809064. 30280641

[ref133] Van Cutsem E.; Huijberts S.; Grothey A.; Yaeger R.; Cuyle P. J.; Elez E.; Fakih M.; Montagut C.; Peeters M.; Yoshino T.; Wasan H.; Desai J.; Ciardiello F.; Gollerkeri A.; Christy-Bittel J.; Maharry K.; Sandor V.; Schellens J. H. M.; Kopetz S.; Tabernero J. Binimetinib, Encorafenib, and Cetuximab Triplet Therapy for Patients With BRAF V600E-Mutant Metastatic Colorectal Cancer: Safety Lead-In Results From the Phase III BEACON Colorectal Cancer Study. J. Clin Oncol 2019, 37 (17), 1460–1469. 10.1200/JCO.18.02459. 30892987 PMC7370699

[ref134] Davis I. D.; Martin A. J.; Stockler M. R.; Begbie S.; Chi K. N.; Chowdhury S.; Coskinas X.; Frydenberg M.; Hague W. E.; Horvath L. G.; Joshua A. M.; Lawrence N. J.; Marx G.; McCaffrey J.; McDermott R.; McJannett M.; North S. A.; Parnis F.; Parulekar W.; Pook D. W.; Reaume M. N.; Sandhu S. K.; Tan A.; Tan T. H.; Thomson A.; Tu E.; Vera-Badillo F.; Williams S. G.; Yip S.; Zhang A. Y.; Zielinski R. R.; Sweeney C. J. Enzalutamide with Standard First-Line Therapy in Metastatic Prostate Cancer. N. Engl. J. Med. 2019, 381 (2), 121–131. 10.1056/NEJMoa1903835. 31157964

[ref135] Rini B. I.; Plimack E. R.; Stus V.; Gafanov R.; Hawkins R.; Nosov D.; Pouliot F.; Alekseev B.; Soulieres D.; Melichar B.; Vynnychenko I.; Kryzhanivska A.; Bondarenko I.; Azevedo S. J.; Borchiellini D.; Szczylik C.; Markus M.; McDermott R. S.; Bedke J.; Tartas S.; Chang Y. H.; Tamada S.; Shou Q.; Perini R. F.; Chen M.; Atkins M. B.; Powles T. Pembrolizumab plus Axitinib versus Sunitinib for Advanced Renal-Cell Carcinoma. N. Engl. J. Med. 2019, 380 (12), 1116–1127. 10.1056/NEJMoa1816714. 30779529

[ref136] Motzer R. J.; Penkov K.; Haanen J.; Rini B.; Albiges L.; Campbell M. T.; Venugopal B.; Kollmannsberger C.; Negrier S.; Uemura M.; Lee J. L.; Vasiliev A.; Miller W. H. Jr.; Gurney H.; Schmidinger M.; Larkin J.; Atkins M. B.; Bedke J.; Alekseev B.; Wang J.; Mariani M.; Robbins P. B.; Chudnovsky A.; Fowst C.; Hariharan S.; Huang B.; di Pietro A.; Choueiri T. K. Avelumab plus Axitinib versus Sunitinib for Advanced Renal-Cell Carcinoma. N Engl J. Med. 2019, 380 (12), 1103–1115. 10.1056/NEJMoa1816047. 30779531 PMC6716603

[ref137] Herrlinger U.; Tzaridis T.; Mack F.; Steinbach J. P.; Schlegel U.; Sabel M.; Hau P.; Kortmann R. D.; Krex D.; Grauer O.; Goldbrunner R.; Schnell O.; Bahr O.; Uhl M.; Seidel C.; Tabatabai G.; Kowalski T.; Ringel F.; Schmidt-Graf F.; Suchorska B.; Brehmer S.; Weyerbrock A.; Renovanz M.; Bullinger L.; Galldiks N.; Vajkoczy P.; Misch M.; Vatter H.; Stuplich M.; Schafer N.; Kebir S.; Weller J.; Schaub C.; Stummer W.; Tonn J. C.; Simon M.; Keil V. C.; Nelles M.; Urbach H.; Coenen M.; Wick W.; Weller M.; Fimmers R.; Schmid M.; Hattingen E.; Pietsch T.; Coch C.; Glas M. Neurooncology Working Group of the German Cancer, S., Lomustine-Temozolomide combination therapy versus standard Temozolomide therapy in patients with newly diagnosed glioblastoma with methylated MGMT promoter (CeTeG/NOA-09): a randomised, open-label, phase 3 trial. Lancet 2019, 393 (10172), 678–688. 10.1016/S0140-6736(18)31791-4. 30782343

[ref138] Fangusaro J.; Onar-Thomas A.; Young Poussaint T.; Wu S.; Ligon A. H.; Lindeman N.; Banerjee A.; Packer R. J.; Kilburn L. B.; Goldman S.; Pollack I. F.; Qaddoumi I.; Jakacki R. I.; Fisher P. G.; Dhall G.; Baxter P.; Kreissman S. G.; Stewart C. F.; Jones D. T. W.; Pfister S. M.; Vezina G.; Stern J. S.; Panigrahy A.; Patay Z.; Tamrazi B.; Jones J. Y.; Haque S. S.; Enterline D. S.; Cha S.; Fisher M. J.; Doyle L. A.; Smith M.; Dunkel I. J.; Fouladi M. Selumetinib in paediatric patients with BRAF-aberrant or neurofibromatosis type 1-associated recurrent, refractory, or progressive low-grade glioma: a multicentre, phase 2 trial. Lancet Oncol 2019, 20 (7), 1011–1022. 10.1016/S1470-2045(19)30277-3. 31151904 PMC6628202

[ref139] Leonard J. P.; Trneny M.; Izutsu K.; Fowler N. H.; Hong X.; Zhu J.; Zhang H.; Offner F.; Scheliga A.; Nowakowski G. S.; Pinto A.; Re F.; Fogliatto L. M.; Scheinberg P.; Flinn I. W.; Moreira C.; Cabecadas J.; Liu D.; Kalambakas S.; Fustier P.; Wu C.; Gribben J. G. AUGMENT: A Phase III Study of Lenalidomide Plus Rituximab Versus Placebo Plus Rituximab in Relapsed or Refractory Indolent Lymphoma. J. Clin Oncol 2019, 37 (14), 1188–1199. 10.1200/JCO.19.00010. 30897038 PMC7035866

[ref140] Winter S. S.; Dunsmore K. P.; Devidas M.; Wood B. L.; Esiashvili N.; Chen Z.; Eisenberg N.; Briegel N.; Hayashi R. J.; Gastier-Foster J. M.; Carroll A. J.; Heerema N. A.; Asselin B. L.; Gaynon P. S.; Borowitz M. J.; Loh M. L.; Rabin K. R.; Raetz E. A.; Zweidler-Mckay P. A.; Winick N. J.; Carroll W. L.; Hunger S. P. Improved Survival for Children and Young Adults With T-Lineage Acute Lymphoblastic Leukemia: Results From the Children’s Oncology Group AALL0434 Methotrexate Randomization. J. Clin Oncol 2018, 36 (29), 2926–2934. 10.1200/JCO.2018.77.7250. 30138085 PMC6366301

[ref141] Katzenstein H. M.; Langham M. R.; Malogolowkin M. H.; Krailo M. D.; Towbin A. J.; McCarville M. B.; Finegold M. J.; Ranganathan S.; Dunn S.; McGahren E. D.; Tiao G. M.; O’Neill A. F.; Qayed M.; Furman W. L.; Xia C.; Rodriguez-Galindo C.; Meyers R. L. Minimal adjuvant chemotherapy for children with hepatoblastoma resected at diagnosis (AHEP0731): a Children’s Oncology Group, multicentre, phase 3 trial. Lancet Oncol 2019, 20 (5), 719–727. 10.1016/S1470-2045(18)30895-7. 30975630 PMC6499702

[ref142] Long G. V.; Saw R. P. M.; Lo S.; Nieweg O. E.; Shannon K. F.; Gonzalez M.; Guminski A.; Lee J. H.; Lee H.; Ferguson P. M.; Rawson R. V.; Wilmott J. S.; Thompson J. F.; Kefford R. F.; Ch’ng S.; Stretch J. R.; Emmett L.; Kapoor R.; Rizos H.; Spillane A. J.; Scolyer R. A.; Menzies A. M. Neoadjuvant dabrafenib combined with trametinib for resectable, stage IIIB-C, BRAF(V600) mutation-positive melanoma (NeoCombi): a single-arm, open-label, single-centre, phase 2 trial. Lancet Oncol 2019, 20 (7), 961–971. 10.1016/S1470-2045(19)30331-6. 31171444

[ref143] Rozeman E. A.; Menzies A. M.; van Akkooi A. C. J.; Adhikari C.; Bierman C.; van de Wiel B. A.; Scolyer R. A.; Krijgsman O.; Sikorska K.; Eriksson H.; Broeks A.; van Thienen J. V.; Guminski A. D.; Acosta A. T.; Ter Meulen S.; Koenen A. M.; Bosch L. J. W.; Shannon K.; Pronk L. M.; Gonzalez M.; Ch’ng S.; Grijpink-Ongering L. G.; Stretch J.; Heijmink S.; van Tinteren H.; Haanen J.; Nieweg O. E.; Klop W. M. C.; Zuur C. L.; Saw R. P. M.; van Houdt W. J.; Peeper D. S.; Spillane A. J.; Hansson J.; Schumacher T. N.; Long G. V.; Blank C. U. Identification of the optimal combination dosing schedule of neoadjuvant ipilimumab plus nivolumab in macroscopic stage III melanoma (OpACIN-neo): a multicentre, phase 2, randomised, controlled trial. Lancet Oncol 2019, 20 (7), 948–960. 10.1016/S1470-2045(19)30151-2. 31160251

[ref144] Dhar R.; Mallik S.; Devi A. Exosomal microRNAs (exoMIRs): micromolecules with macro impact in oral cancer. 3 Biotech 2022, 12 (7), 155 10.1007/s13205-022-03217-z. PMC9234016 35769549

[ref145] Li Y. J.; Wu J. Y.; Wang J. M.; Hu X. B.; Cai J. X.; Xiang D. X. Gemcitabine loaded autologous exosomes for effective and safe chemotherapy of pancreatic cancer. Acta Biomater. 2020, 101, 519–530. 10.1016/j.actbio.2019.10.022. 31629893

[ref146] Smyth T.; Kullberg M.; Malik N.; Smith-Jones P.; Graner M. W.; Anchordoquy T. J. Biodistribution and delivery efficiency of unmodified tumor-derived exosomes. J. Controlled Release 2015, 199, 145–155. 10.1016/j.jconrel.2014.12.013. PMC4441346 25523519

[ref147] Imai T.; Takahashi Y.; Nishikawa M.; Kato K.; Morishita M.; Yamashita T.; Matsumoto A.; Charoenviriyakul C.; Takakura Y. Macrophage-dependent clearance of systemically administered B16BL6-derived exosomes from the blood circulation in mice. J. Extracell. Vesicles 2015, 4 (1), 26238 10.3402/jev.v4.26238. 25669322 PMC4323410

[ref148] Kooijmans S.A.A.; Fliervoet L.A.L.; van der Meel R.; Fens M.H.A.M.; Heijnen H.F.G.; van Bergen en Henegouwen P.M.P.; Vader P.; Schiffelers R.M. PEGylated and targeted extracellular vesicles display enhanced cell specificity and circulation time. J. Controlled Release 2016, 224, 77–85. 10.1016/j.jconrel.2016.01.009. 26773767

[ref149] Shivji G. G.; Dhar R.; Devi A. Role of exosomes and its emerging therapeutic applications in the pathophysiology of non-infectious diseases. Biomarkers 2022, 27 (6), 534–548. 10.1080/1354750X.2022.2067233. 35451890

[ref150] Dhar R.; Mukherjee S.; Mukerjee N.; Mukherjee D.; Devi A.; Ashraf G. M.; Alserihi R. F.; Tayeb H. H.; Hashem A. M.; Alexiou A.; Thorate N. Interrelation between extracellular vesicles miRNAs with chronic lung diseases. J. Cell Physiol. 2022, 237 (11), 4021–4036. 10.1002/jcp.30867. 36063496

[ref151] Wang J.; Chen D.; Ho E. A. Challenges in the development and establishment of exosome-based drug delivery systems. J. Controlled Release 2021, 329, 894–906. 10.1016/j.jconrel.2020.10.020. 33058934

[ref152] Kim H.; Jang H.; Cho H.; Choi J.; Hwang K. Y.; Choi Y.; Kim S. H.; Yang Y. Recent Advances in Exosome-Based Drug Delivery for Cancer Therapy. Cancers (Basel) 2021, 13 (17), 4435 10.3390/cancers13174435. 34503245 PMC8430743

[ref153] Jang S. C.; Kim O. Y.; Yoon C. M.; Choi D. S.; Roh T. Y.; Park J.; Nilsson J.; Lötvall J.; Kim Y. K.; Gho Y. S. Bioinspired exosome-mimetic nanovesicles for targeted delivery of chemotherapeutics to malignant tumors. ACS Nano 2013, 7 (9), 7698–710. 10.1021/nn402232g. 24004438

[ref154] Adriano B.; Cotto N. M.; Chauhan N.; Jaggi M.; Chauhan S. C.; Yallapu M. M. Milk exosomes: Nature’s abundant nanoplatform for theranostic applications. Bioact Mater. 2021, 6 (8), 2479–2490. 10.1016/j.bioactmat.2021.01.009. 33553829 PMC7856328

[ref155] Dhar R.; Gorai S.; Krishnan A.; Mukherjee D. Why India needs more exosome research for cancer?. Ann. Med. Surg. 2022, 80, 104265 10.1016/j.amsu.2022.104265. PMC9358430 35958283

[ref156] Dhar R.; Mukherjee D.; Mukerjee N.; Devi A.; Dey A.; Ghosh A. Exosome based theranostic approaches in breast cancer, a new answer of Indian breast cancer-associated health crisis - Correspondence. Int. J. Surg 2022, 105, 106886 10.1016/j.ijsu.2022.106886. 36084809

[ref157] Dhar R.; Bhattacharya B.; Mandal D.; Devi A.; Thorat N. D. Exosome-based cancer vaccine: A cutting-edge approach - Correspondence. Int. J. Surg 2022, 108, 106993 10.1016/j.ijsu.2022.106993. 36356827

[ref158] Krishnan A.; Bhattacharya B.; Mandal D.; Dhar R.; Muthu S. Salivary exosomes: A theranostics secret of oral cancer - Correspondence. Int. J. Surg 2022, 108, 106990 10.1016/j.ijsu.2022.106990. 36368419

[ref159] Hoque S.; Dhar R.; Kar R.; Mukherjee S.; Mukherjee D.; Mukerjee N.; Nag S.; Tomar N.; Mallik S. Cancer Stem Cells (CSCs): key player of radiotherapy resistance and its clinical significance. Biomarkers 2022, 1–28. 10.1080/1354750X.2022.2157875. 36503350

